# Coupling finite volume-lattice Boltzmann methods for advanced heat transfer simulations

**DOI:** 10.1007/s00366-026-02288-3

**Published:** 2026-02-26

**Authors:** Yang Zhou, Alessandro De Rosis, Alistair Revell

**Affiliations:** Department of Mechanical and Aerospace Engineering, The University of Manchester, Manchester, M13 9PL UK

**Keywords:** CFD, Coupled simulations, Thermal flows, Lattice Boltzmann method

## Abstract

We present a high-performance coupled framework that advances the integration of the finite volume method (FVM) and the lattice Boltzmann method (LBM) for multi-physics thermal flow simulations, including heat conduction, conjugated heat transfer, natural and forced convection, and phase change. The proposed scheme employs a central-moments-based collision operator for both velocity and temperature fields, substantially improving numerical stability and accuracy over traditional approaches within the LBM community. The reconstruction strategy, combining regularised and high-order truncated equilibrium methods, ensures smooth and accurate data exchange at FVM–LBM coupling interfaces. The implementation employs the Parallel Location and Exchange coupling library, enabling efficient and scalable communication between the FVM and LBM. Validation against standard benchmark problems and complex melting scenarios demonstrates excellent numerical accuracy and convergence. These algorithmic advances establish the proposed framework as a significant step forward in coupled FVM-LBM methods for multiscale thermal flow problems.

## Introduction

Multiscale thermo-fluid problems, characterised by disparate spatial and temporal scales, are widespread in practical engineering systems such as heat exchangers [1], phase-change devices and fuel cells [2, 3]. Accurately and efficiently predicting such multiscale phenomena remains a significant challenge for conventional monolithic simulation approaches, which often struggle to balance computational efficiency with physical fidelity [4]. These limitations have motivated the development of advanced coupled numerical framework for multiscale simulations, in which the broader system behaviour is captured at macroscopic level with a comprehensive but less detailed view, while the finer-scale physics are resolved using meso/microscopic methods that are imperceptible at the macroscopic resolution. Such coupling strategies enhance both physical insight and predictive capability without the prohibitive computational demands of a purely microscopic approach.

Coupling strategies for thermo-fluid simulations generally fall into two main paradigms. The first applies heterogeneous numerical methods across the entire computational domain, assigning different techniques to distinct physical fields and coupling them through macroscopic governing equations [5–8]. The second adopts a domain decomposition approach, dividing the domain into subregions, each solved by a different numerical method [9–11], with key variables (such as velocity and temperature) exchanged at the interfaces. This latter approach is particularly well-suited to multiscale problems, as it enables tailored spatial and temporal resolutions in different regions. Indeed, macroscopic solvers efficiently handle large-scale domains with broader but coarser representations, while meso/microscopic methods resolve fine-scale details within localised regions that would otherwise be lost. This synergistic integration strikes a crucial balance, achieving both the computational efficiency necessary for large-scale simulations and the physical fidelity required to capture the multiscale complexity of real-world thermo-fluid systems.

In response to their practical importance, considerable researches have focused on developing coupled numerical frameworks for multiscale thermo-fluid simulations. A number of these coupling schemes have been successfully established and validated through rigorous benchmark studies, including hybrid combinations such as molecular dynamics–finite volume method [12, 13], molecular dynamics–lattice Boltzmann method [14], direct simulation Monte Carlo–finite volume method [15], lattice Boltzmann method–finite difference method [16], and finite volume method–lattice Boltzmann method (FVM–LBM) [9, 10]. Amongst available methods, the lattice Boltzmann method (LBM) stands out as a powerful mesoscopic approach, bridging the gap between microscopic and macroscopic models for fluid flow and heat transfer. In short, the LBM computes the spatio-temporal evolution of collections (also known as populations or distributions) of fictitious particles, which carry information about the underlying flow physics. In parallel, the finite volume method (FVM) remains the leading choice for macroscopic simulations, valued for its robustness and computational efficiency in engineering applications. By combining these strengths, the coupled FVM–LBM framework offers a compelling solution for practical multiscale problems, capturing fine microscale physics with LBM while leveraging FVM’s ability to efficiently handle large-scale domains.

A central challenge in FVM–LBM coupling lies in the accurate reconstruction of LBM particle distribution functions from macroscopic variables, due to the lack of a direct mapping between the two formulations. This issue was first addressed by Xu et al. [9], who derived analytical relations between macroscopic fields and mesoscopic populations using a single-relaxation-time (SRT) LBM scheme based on the Bhatnagar-Gross-Krook (BGK) approximation. Their pioneering framework enabled coupled simulations of laminar lid-driven cavity flows up to Reynolds numbers of 1000. Building on this foundation, Luan et al. [17, 18] extended the methodology to more complex configurations, including airfoil aerodynamics and porous media transport, thereby highlighting the framework’s versatility for multiscale flow problems. Later, Tong et al. [19] applied the FVM–LBM coupling to model fouling layer formation on heat exchanger tubes, validating the approach for industrial-scale particulate flows through parametric studies on particle size and inlet velocity.

Efforts to extend coupled FVM–LBM methods for thermal flows have also seen important progress [20]. Luan et al. [21] developed the first thermal coupling algorithm based on Chapman–Enskog expansion under the SRT scheme, and validated it in two-dimensional natural convection scenarios. Tong & He [22] proposed a unified FVM–LBM framework capable of simulating coupled flow and heat transfer processes up to Rayleigh numbers (Ra) of $1.4\times 10^5$. Salimi et al. [11] improved numerical stability by incorporating a multiple-relaxation-time (MRT) collision operator, though their reconstruction operators remained based on SRT LBM [21]. Their work also explored conjugate heat transfer over heated square cylinders with porous layers. At the cost of increased complexity, Salimi et al. [23] developed reconstruction operators tailored to the MRT framework and demonstrated successful simulations of three-dimensional natural convection at Ra = $10^5$. Alternative approaches used D2Q9 and D2Q5 lattice models with non-equilibrium extrapolation for velocity and temperature fields, respectively [10]. However, simulations at Ra = $10^6$ still exhibited interfacial temperature discontinuities, highlighting unresolved challenges in high-Rayleigh-number coupled simulations.

Despite significant progress, three major challenges still limit current FVM–LBM coupling strategies for thermal flows. First, reconstruction algorithms often involve high computational costs, reducing the overall efficiency and practical applicability of the coupled schemes. Second, validation remains limited for complex regimes such as high-Rayleigh-number natural convection, forced convection, and conjugate heat transfer. Third, little attention has been given to large-scale parallel implementations, which are crucial for industrial applications, as highlighted by He & Tao [24].

To address these gaps, here we develop a robust two-way coupling framework integrating the industrial FVM solver $\textit{code\_saturne}$ [25] and the LBM solver LUMA [26], facilitated by the Parallel Location and Exchange (PLE) library [27] for efficient data transfer. This framework provides proof-of-concept applications for multiscale thermal flow simulations, in which the mesoscopic LBM resolves the local thermal flow in one sub-domain while the macroscopic FVM is used to simulate the another sub-domain. Building upon the multiphysics groundwork by De Rosis & Coreixas [28], we advance the state-of-the-art by implementing a thermal LBM scheme based on a central-moments (CMs) collision operator. This CMs-based scheme offers enhanced stability and accuracy compared to traditional MRT and SRT models [29–32]. Additionally, we adopt regularised and high-order truncated equilibrium distribution schemes to reconstruct the particle distribution functions from macroscopic data [4, 33]. The framework is rigorously validated across several well-consolidated benchmark cases involving natural and forced convection, as well as conjugate heat transfer. Moreover, it proves particularly effective in capturing complex melting dynamics, benefiting from the complementary strengths of the FVM and LBM approaches.

The rest of this paper is organised as follows. Section 2 presents the numerical methodology underpinning the coupling framework. Section 3 evaluates its performance across a suite of benchmark problems. Section 4 concludes with key findings and outlines future research directions in FVM–LBM coupling for thermal flow simulations.

## Methodology

### Governing equations

Let us consider a three-dimensional Cartesian coordinate system of axes $x-y-z$. The macroscopic behaviour of an incompressible Newtonian fluid with thermal effects (under the Boussinesq approximation) is governed by the following set of equations:

1 $$\begin{aligned} \displaystyle \boldsymbol{\nabla } \cdot \boldsymbol{u}= & 0 , \end{aligned}$$

2 $$\begin{aligned} &\displaystyle \partial _t \boldsymbol{u} + \left( \boldsymbol{u} \cdot \boldsymbol{\nabla } \right) \boldsymbol{u}= -\dfrac{1}{\rho _0} \boldsymbol{\nabla } p \\&+ \nu \boldsymbol{\nabla }^2 \boldsymbol{u} +\boldsymbol{g} \beta \left( T-T_0\right) , \end{aligned}$$

3 $$\begin{aligned} {\partial _t T} + \boldsymbol{u} \cdot \boldsymbol{\nabla } T= & \boldsymbol{\nabla } \cdot (\alpha \boldsymbol{\nabla } T), \end{aligned}$$

where $\boldsymbol{u}=[u_x, u_y, u_z]$ denotes the velocity vector, *t* is the time, $\rho _0$ is the reference mass fluid density, *p* indicates the pressure, $\nu$ denotes the fluid kinematic viscosity, *T* denotes the temperature, the thermal diffusivity is defined as $\alpha = {\lambda /( \rho c_p})$, with $\lambda$ and $c_p$ representing the thermal conductivity and specific heat capacity of the fluid, respectively, $T_0$ is the reference temperature, and $\beta$ represents the thermal expansion coefficient. The acceleration due to gravity $\boldsymbol{g}$ acts in the direction *y* and has module $g=-9.806$. Gradient and Laplacian operators are defined as $\boldsymbol{\nabla } = [\partial _x, \partial _y, \partial _z]$ and $\boldsymbol{\nabla }^2 = [\partial ^2_x+\partial ^2_y+\partial ^2_z]$, respectively, $\partial$ representing a partial derivative.

The physics of the problem is governed by the Rayleigh number and the Prandtl number, which are defined as

4 $$\begin{aligned} \textrm{Ra} = \frac{g \, \beta \, \Delta T \, L^3}{\nu \, \alpha }, \qquad \textrm{Pr} = \frac{\nu }{\alpha }, \end{aligned}$$

where *L* and $\Delta T$ are the characteristic length and temperature difference of the problem, respectively.

In the FVM implementation, the computational domain is decomposed into numerous control volumes. The governing Eqs. (1)–(3) are numerically integrated over each control volume to derive the corresponding discretised equations. For resolving the velocity-pressure coupling, the semi-implicit method for pressure-linked equation consistent [25] algorithm is employed on the FVM side.

Unlike traditional Navier–Stokes solvers, which operate at the macroscopic scale, the lattice Boltzmann method models fluid dynamics from a mesoscopic perspective. It tracks the spatio-temporal evolution of discrete particle distribution functions, whose statistical moments yield macroscopic quantities such as velocity and pressure. For thermal flow simulations, we adopt the double-distribution function (DDF) approach, which employs two distinct sets of distribution functions: one for the velocity field and another for the temperature field. This separation allows for efficient and accurate resolution of coupled thermo-fluid dynamics within the LBM framework. Let us consider the classic BGK scheme with D3Q19 spatial discretization first. The lattice Boltzmann equation (LBE) for the velocity and temperature fields are defined as

5 $$\begin{aligned} &\displaystyle f_i(\boldsymbol{x} + \boldsymbol{c}_i \Delta t, t + \Delta t)= f_i(\boldsymbol{x}, t)\\&+ \Omega _i(\boldsymbol{x},t) + \left( 1-{\omega \over 2}\right) F_i(\boldsymbol{x}, t), \end{aligned}$$

6 $$\begin{aligned} g_i(\boldsymbol{x} + \boldsymbol{c}_i \Delta t, t + \Delta t)&= g_i(\boldsymbol{x}, t)+ \Omega _{T,i}(\boldsymbol{x},t), \end{aligned}$$

where $\boldsymbol{x}=[x, \, y, \, z]$ represents the spatial coordinates of a lattice node, the time step $\Delta t$ is implicitly taken as 1. The particle distribution functions $|f_i\rangle = [f_0,...,~f_{i},...,~f_{18}]^{\top }$ and $|g_i\rangle = [g_0,...,~g_{i},...,~g_{18}]^{\top }$ govern the evolution of the velocity and temperature fields, respectively, with the index $i=0,\,1,..., \,17,\,18$ representing the discrete velocity directions in the D3Q19 model. Here and henceforth, the Dirac notation $|\bullet \rangle$ indicates a column vector and the $\top$ represents the transpose operator. These distribution functions propagate along the prescribed lattice directions $\boldsymbol{c}_i = \left[ |c_{ix}\rangle ,~|c_{iy}\rangle ,~|c_{iz}\rangle \right]$, which are defined in Eq. (11). Within the BGK approximation, the collision operators $\Omega _i$ and $\Omega _{T,i}$ can be written as a relaxation of populations towards an equilibrium state,

7 $$\begin{aligned} \Omega _i= \omega (f_i^{eq}-f_i), \end{aligned}$$

8 $$\begin{aligned} \Omega _{T,i}= \omega _T (g_i^{eq}-g_i), \end{aligned}$$

where the superscript *eq* denotes the equilibrium distribution functions. The macroscopic governing Eqs. (1)–(3) are recovered through Chapman-Enskog analysis [34], where the kinematic viscosity $\nu$ and thermal diffusivity $\alpha$ are related to the relaxation frequency $\omega$ and $\omega _T$, respectively, through the expressions

9 $$\begin{aligned} \nu= & c_s^2\left( 1/\omega - {1 / 2}\right) , \end{aligned}$$

10 $$\begin{aligned} \alpha= & c_s^2\left( 1/\omega _T - {1/ 2}\right) , \end{aligned}$$

where $c_s=1/\sqrt{3}$ represents the lattice sound speed in the D3Q19 discretisation scheme. While the source term $F_i$ is commonly handled through the second-order truncated model of Guo et al. [35], in this work we instead adopt the fully Hermite-based formulation introduced by De Rosis & Coreixas [28]. In addition, the equilibrium distribution functions $f_i^{eq}$ and $g_i^{eq}$ in the CMs-based LBM scheme are not approximated using the conventional second-order truncated formulation, and the incompressibility correction is not employed in the present study. In fact, exploiting the full potential of any lattice Boltzmann discretisation requires the usage of the complete allowable set of Hermite polynomials [36, 37]. Defining a tensor product notation for the discrete parameter space $(\psi ,\gamma ,\chi )\in \{\pm 1\}^3$ and making reference to De Rosis & Coreixas [28], $f_i^{eq}$ and $g_i^{eq}$ are rewritten in Eq. (12)

11 $$\begin{aligned} | c_{ix}\rangle&= [0,\, 1,\, -1,\, 0,\, 0,\, 0,\, 0,\, 1,\, \nonumber \\&-1,\, 1,\, -1,\, 1,\, -1,\, 1,\, -1,\, 0,\, 0,\, 0,\, 0 ]^{\top }, \nonumber \\ | c_{iy}\rangle&= [0,\, 0,\, 0,\, 1,\, \nonumber \\&-1,\, 0,\, 0,\, 1,\, -1,\, -1,\, 1,\, 0,\, 0,\, 0,\, 0,\, 1,\, -1,\, 1,\, -1 ]^{\top }, \nonumber \\ | c_{iz}\rangle&= [0,\, 0,\, 0,\, 0,\, 0,\, 1,\, -1,\, 0,\, 0,\, 0, \, 0,\, 1,\, \nonumber \\&-1,\, -1,\, 1,\, 1,\, -1,\, -1,\, 1 ]^{\top }. \end{aligned}$$

12 $$\begin{aligned} & f^{eq}_{(0, 0, 0)}=g^{eq}_{(0, 0, 0)}=\frac{\xi }{3} \left[ 1-(u_x^2+u_y^2+u_z^2)\right. \nonumber \\ & \left. + 3(u_x^2 u_y^2+u_x^2 u_z^2+u_y^2 u_z^2)\right] ,\nonumber \\ & f^{eq}_{(\psi , 0, 0)}=g^{eq}_{(\psi , 0, 0)}= \frac{\xi }{18} \left[ 1 +3 \psi u_x + 3 (u_x^2- u_y^2- u_z^2) \right. \nonumber \\ & -\left. 9\psi (u_x u_y^2 + u_x u_z^2)-9 (u_x^2 u_y^2 + u_x^2 u_z^2)\right] ,\nonumber \\ & f^{eq}_{(0, \lambda , 0)}=g^{eq}_{(0, \lambda , 0)}= \frac{\xi }{18} \left[ 1 +3 \lambda u_y +3 (- u_x^2+ u_y^2- u_z^2) \right. \nonumber \\ & \left. -9\lambda (u_x^2 u_y + u_y u_z^2)-9 (u_x^2 u_y^2+ u_y^2 u_z^2)\right] ,\nonumber \\ & f^{eq}_{(0, 0, \chi )}=g^{eq}_{(0, 0, \chi )}= \frac{\xi }{18} \left[ 1+3 \chi u_z +3(- u_x^2- u_y^2+ u_z^2) \right. \nonumber \\ & \left. -9\chi (u_x^2 u_z + u_y^2 u_z)-9 (u_x^2 u_z^2+ u_y^2 u_z^2)\right] , \\ & f^{eq}_{(\psi , \lambda , 0)}=g^{eq}_{(\psi , \lambda , 0)} = \frac{\xi }{36} \left[ 1+3(\psi u_x + \lambda u_y) + 3 (u_x^2+ u_y^2)+9\psi \lambda u_x u_y\right. \nonumber \\ & \left. + 9(\lambda u_x^2 u_y+\psi u_x u_y^2)+9 u_x^2 u_y^2\right] ,\nonumber \\ & f^{eq}_{(\psi , 0, \chi )}=g^{eq}_{(\psi , 0, \chi )} = \frac{\xi }{36} \left[ 1+3(\psi u_x + \chi u_z) + 3 (u_x^2+ u_z^2) \right. \nonumber \\ & \left. +9\psi \chi u_x u_z+ 9(\chi u_x^2 u_z+\psi u_x u_z^2)+9 u_x^2 u_z^2\right] ,\nonumber \\ & f^{eq}_{(0, \lambda , \chi )}=g^{eq}_{(0, \lambda , \chi )}= \frac{\xi }{36} \left[ 1+3(\lambda u_y + \chi u_z) + 3 (u_y^2+ u_z^2)\right. \nonumber \\ & \left. +9\lambda \chi u_y u_z+ 9(\chi u_y^2 u_z+\lambda u_y u_z^2)+9 u_y^2 u_z^2\right] ,\nonumber \end{aligned}$$

where $\xi$ equals to the value of density ($\rho$) and temperature (*T*) for the velocity and temperature fields, respectively. The above equilibrium states are able to recover certain third- and fourth-order moments of the Maxwell-Boltzmann distribution. While fourth-order equilibrium moments do not affect the macroscopic behaviour but only on the numerical stability [38], the third-order moments are essential for recovering the correct viscous stress tensor [37]. Eventually, the macroscopic variables ($\rho$, $\boldsymbol{u}$ and *T*) are obtained by

13 $$\begin{aligned} \rho (\boldsymbol{x},t)= & \sum _{i=0}^{18} f_i(\boldsymbol{x}, t), \end{aligned}$$

14 $$\begin{aligned} \boldsymbol{u}(\boldsymbol{x},t)= & \frac{1}{\rho (\boldsymbol{x},t)} \left[ \sum _{i=0}^{18} \boldsymbol{c}_i f_i(\boldsymbol{x}, t) + {\boldsymbol{F}(\boldsymbol{x},t) \over 2} \right] , \end{aligned}$$

15 $$\begin{aligned} T(\boldsymbol{x},t)= & \sum _{i=0}^{18} g_i(\boldsymbol{x}, t). \end{aligned}$$

where $\displaystyle \boldsymbol{F}(\boldsymbol{x},t) = \rho (\boldsymbol{x},t) \, \boldsymbol{g} \, \beta \, \left[ T(\boldsymbol{x},t)-T_0 \right]$ for buoyancy force induced by temperature difference.

### Central-moments-based LBM for velocity field

When implementing the central-moments-based (CMs) LBM collision operator for fluid flow simulations, the lattice Boltzmann equation (LBE) can be expressed as

16 $$\begin{aligned} &\displaystyle |f_i(\boldsymbol{x} + \boldsymbol{c}_i, t + 1)\rangle= |f_i(\boldsymbol{x}, t)\rangle \\&+ \boldsymbol{\Lambda } \left( |f_i^{eq}(\boldsymbol{x}, t)\rangle - |f_i(\boldsymbol{x}, t)\rangle \right) \nonumber \\&+ (\boldsymbol{\textrm{I}} - \boldsymbol{\Lambda }/2)|F_i(\boldsymbol{x}, t)\rangle , \end{aligned}$$

where $\textbf{I}$ is the $19 \times 19$ unit tensor, $\boldsymbol{\Lambda }$ denotes the $19 \times 19$ collision matrix [28, 39], and its role will be elucidated later. The term $F_i$ accounts for external body forces $\boldsymbol{F}=[F_x,F_y,F_z]$, and its prefactor accounts for discrete effects originating from the change of variables that aims at obtaining a numerical scheme explicit in time [35]. The LBE can be split into the collision and streaming steps, which are expressed as

17 $$\begin{aligned}&|f_i^\star (\boldsymbol{x}, t)\rangle = |f_i(\boldsymbol{x}, t)\rangle + \boldsymbol{\Lambda } \left( |f_i^{eq}(\boldsymbol{x}, t)\rangle - |f_i(\boldsymbol{x}, t)\rangle \right) \nonumber \\&\qquad \qquad + (\boldsymbol{\textrm{I}} - \boldsymbol{\Lambda }/2)|F_i(\boldsymbol{x}, t)\rangle , \end{aligned}$$

18 $$\begin{aligned}&|f_i(\boldsymbol{x}+\boldsymbol{c}_i, t+1)\rangle = |f_i^\star (\boldsymbol{x}, t)\rangle , \end{aligned}$$

where the superscript $\star$ represents the post-collision distribution functions. For a CM-based collision operator, the discrete velocities $\displaystyle \boldsymbol{\overline{c}}_i = \left[ \langle \overline{c}_{ix}|, \langle \overline{c}_{iy}|, \langle \overline{c}_{iz}| \right] ^{\top }$ are shifted by the local fluid velocity [29], which are defined as

19 $$\begin{aligned} \langle \overline{c}_{ix}|= & \langle c_{ix} - u_x|, \nonumber \\ \langle \overline{c}_{iy}|= & \langle c_{iy} - u_y|, \nonumber \\ \langle \overline{c}_{iz}|= & \langle c_{iz} - u_z|. \end{aligned}$$

where the symbol $\langle \bullet |$ represents a row vector. To apply the collision step in the CMs space, a suitable basis is used to transform CMs from populations, and transformation matrix $\textbf{T}$ is given by  [28]

20 $$\begin{aligned} {\textbf{T}} = \left[ \begin{array}{c} \langle |\boldsymbol{c}_i|^0| \\ \langle \bar{c}_{ix}|\\ \langle \bar{c}_{iy}| \\ \langle \bar{c}_{iz}| \\ \langle \bar{c}_{ix}^2+ \bar{c}_{iy}^2 + \bar{c}_{iz}^2 | \\ \langle \bar{c}_{ix}^2- \bar{c}_{iy}^2 | \\ \langle \bar{c}_{iy}^2- \bar{c}_{iz}^2|\\ \langle \bar{c}_{ix} \bar{c}_{iy}| \\ \langle \bar{c}_{ix} \bar{c}_{iz} | \\ \langle \bar{c}_{iy} \bar{c}_{iz}| \\ \langle \bar{c}_{ix}^2\bar{c}_{iy}|\\ \langle \bar{c}_{ix}\bar{c}_{iy}^2| \\ \langle \bar{c}_{ix}^2\bar{c}_{iz}|\\ \langle \bar{c}_{ix}\bar{c}_{iz}^2| \\ \langle \bar{c}_{iy}^2\bar{c}_{iz}| \\ \langle \bar{c}_{iy}\bar{c}_{iz}^2| \\ \langle \bar{c}_{ix}^2\bar{c}_{iy}^2| \\ \langle \bar{c}_{ix}^2\bar{c}_{iz}^2| \\ \langle \bar{c}_{iy}^2\bar{c}_{iy}^2| \end{array} \right] . \end{aligned}$$

The collision operator in the populations space is given by $\mathbf {\Lambda } = \mathbf {T^{-1} K T}$, where in the present study $\textbf{K}=\textrm{diag}[1,~1,~1,~1,~1,$ $\omega ,~\omega ,~\omega ,~\omega ,~\omega ,$ $1,~1,~1,~1,~1,~1,~1,~1,~1]$ is the $19\times 19$ relaxation matrix in the CM space while the omitted elements are equal to zero. Notably, if the main diagonal elements of the relaxation matrix are replaced with the relaxation frequency ($\omega$), the CM scheme will transform into CM-SRT scheme [40]. Substituting the relaxation matrix $\mathbf {\Lambda }$ into collision process in Eq. (17), and it takes place as

21 $$\begin{aligned} |k_i^*\rangle =(\mathbf {I-K})|k_i\rangle + \textbf{K}|k_i^{eq}\rangle + \left( \textbf{I} - {\textbf{K}\over 2} \right) |R_i\rangle . \end{aligned}$$

where the post-collision, pre-collision, equilibrium and forcing term CMs are calculated by $|k_i^{*}\rangle = \textbf{T}|f_i^*\rangle$, $|k_i\rangle = \textbf{T}|f_i\rangle$, $|k_i^{eq}\rangle = \textbf{T}|f_i^{eq}\rangle$ and $|R_i\rangle = \textbf{T}|F_i\rangle$, respectively. Applying the transformation matrix $\textbf{T}$ to equilibrium populations in Eq. (12) generates the equilibrium CMs

22 $$\begin{aligned} & k_0^{eq}=\rho , \nonumber \\ & k_4^{eq}=3\rho c_s^2, \nonumber \\ & k_{16}^{eq}=\rho c_s^4,\\ & k_{17}^{eq}=\rho c_s^4, \nonumber \\ & k_{18}^{eq}=\rho c_s^4,\nonumber \end{aligned}$$

while the remaining terms are equal to zero. After the collision, non-zero CMs obtain as [28]

23 $$\begin{aligned} & k_0^*=\rho ,\nonumber \\ & k_1^*=F_x/2,\nonumber \\ & k_2^*=F_y/2,\nonumber \\ & k_3^*=F_z/2,\nonumber \\ & k_4^*=3\rho c_s^2,\nonumber \\ & k_5^*=(1-\omega ) k_5,\nonumber \\ & k_6^*=(1-\omega ) k_6,\nonumber \\ & k_7^*=(1-\omega ) k_7,\nonumber \\ & k_8^*=(1-\omega ) k_8,\\ & k_9^*=(1-\omega ) k_9,\nonumber \\ & k_{10}^*=k_{15}^*=F_y c_s^2/2,\nonumber \\ & k_{11}^*=k_{13}^*=F_x c_s^2/2,\nonumber \\ & k_{12}^*=k_{14}^*=F_z c_s^2/2,\nonumber \\ & k_{16}^*=\rho c_s^4,\nonumber \\ & k_{17}^*=\rho c_s^4,\nonumber \\ & k_{18}^*=\rho c_s^4,\nonumber \end{aligned}$$

where pre-collision CMs are given by

24 $$\begin{aligned} & k_5=\sum _i^{18}f_i(\overline{c}_{ix}^2-\overline{c}_{iy}^2), \nonumber \\ & k_6=\sum _i^{18}f_i(\overline{c}_{iy}^2-\overline{c}_{iz}^2), \nonumber \\ & k_7=\sum _i^{18}f_i \overline{c}_{ix} \overline{c}_{iy}, \\ & k_8=\sum _i^{18}f_i \overline{c}_{ix} \overline{c}_{iz}, \nonumber \\ & k_9=\sum _i^{18}f_i \overline{c}_{iy} \overline{c}_{iz}. \nonumber \end{aligned}$$

The post-collision populations can be obtained by

25 $$\begin{aligned} |f^*_i\rangle =\textbf{T}^{-1}|k_i^*\rangle . \end{aligned}$$

According to the Refs. [41, 42], the two-step approach is adopted to compute the post-collision distribution function from central moments. Eventually, the populations are streamed following the directions in Eq. (11) and the macroscopic density and velocity are obtained by Eqs. (13)–(14).

### Central-moments-based LBM for temperature field

Within the CMs-based LBM framework, the LBE for the convection-diffusion process in Eq. (3) without heat source contributions can be formulated as

26 $$\begin{aligned} |g_i(\boldsymbol{x}+\boldsymbol{c}_i, t+1)\rangle = |g_i(\boldsymbol{x},t)\rangle + \boldsymbol{\Lambda }_T \left( |g_i^{eq}(\boldsymbol{x},t)\rangle -|g_i(\boldsymbol{x},t)\rangle \right) , \end{aligned}$$

where $\boldsymbol{\Lambda }_T$ represents the $19 \times 19$ collision matrix for temperature distribution functions. This thermal LBE is decomposed into the collision and streaming steps as

27 $$\begin{aligned} & |g_i^{\star }(\boldsymbol{x},t)\rangle = |g_i(\boldsymbol{x},t)\rangle + \boldsymbol{\Lambda }_T \left( |g_i^{eq}(\boldsymbol{x},t)\rangle -|g_i(\boldsymbol{x},t)\rangle \right) , \end{aligned}$$

28 $$\begin{aligned} & |g_i(\boldsymbol{x}+\boldsymbol{c}_i, t+1)\rangle = |g_i^{\star }(\boldsymbol{x},t)\rangle . \end{aligned}$$

For the thermal CM-based scheme, the lattice directions are also shifted by the local fluid velocity through the Eqs. (19). In addition, the collision matrix is formulated as $\boldsymbol{\Lambda }_T = \textbf{T}^{-1} \textbf{K}_T \textbf{T}$, where the same transformation matrix $\textbf{T}$ in Eq. (20) is applied to the collision step. $\textbf{K}_T$ represents the diagonal matrix of relaxation rates in temperature field, which is defined as $\textbf{K}_T=\textrm{diag}$ $[1,~\omega _T,~\omega _T,~\omega _T,$ $1,~1,~1,~1,~1,~1,~1,~1,~1,$ $~1,~1,~1,~1,~1,~1]$, and the off-diagonal elements remain as zero.

The collision operation is executed in the space of CMs, and it is expressed as

29 $$\begin{aligned} {\begin{matrix} |k_{i,T}^{\star }\rangle =& \left( \textbf{I}- \textbf{K}_{T} \right) \textbf{T} |g_i\rangle + \textbf{K}_{T} \textbf{T} |g_i^{\textrm{eq}}\rangle \\ =& \left( \textbf{I}- \textbf{K}_{T} \right) |k_{i,T}\rangle + \textbf{K}_{T} |k_{i_,T}^{\textrm{eq}}\rangle , \end{matrix}} \end{aligned}$$

where the vectors $|k_{i,T}\rangle$, $|k_{i,T}^{\textrm{eq}}\rangle$ and $|k_{i,T}^{\star }\rangle$ represent the collections of pre-collision, equilibrium and post-collision moments in temperature field, respectively. Following the definition $|k_{i,T}^{\textrm{eq}}\rangle = \textbf{T} |g_i^{\textrm{eq}}\rangle$, only five equilibrium moments are non-zero, i.e.,

30 $$\begin{aligned} k_{0,T}^{eq}= & T, \nonumber \\ k_{4,T}^{eq}= & T, \nonumber \\ k_{16,T}^{eq}= & T c_{s}^4, \nonumber \\ k_{17,T}^{eq}= & T c_{s}^4, \nonumber \\ k_{18,T}^{eq}= & T c_{s}^4. \end{aligned}$$

Moreover, the equilibrium moments exhibit both elegant mathematical form and Galilean invariance, that is, they remain independent of the reference frame velocity. Such findings align with the theoretical analysis carried out by De Rosis & Luo [31], where they demonstrated that Galilean invariant equilibrium central moments can be obtained only when the transformation matrix is applied to equilibrium populations equipped with the complete basis of Hermite polynomials. The non-zero post-collision central moments are given by

31 $$\begin{aligned} k_{0,T}^{\star }= & T, \nonumber \\ k_{4,T}^{\star }= & T, \nonumber \\ k_{1,T}^{\star }= & \left( 1-\omega _{T} \right) k_{1,T}, \nonumber \\ k_{2,T}^{\star }= & \left( 1-\omega _{T} \right) k_{2,T}, \nonumber \\ k_{3,T}^{\star }= & \left( 1-\omega _{T} \right) k_{3,T}, \nonumber \\ k_{16,T}^{\star }= & T c_{s}^4,\nonumber \\ k_{17,T}^{\star }= & T c_{s}^4,\nonumber \\ k_{18,T}^{\star }= & T c_{s}^4, \end{aligned}$$

where the required pre-collision central moments are

32 $$\begin{aligned} k_{1,T}= & \sum _{i=0}^{18} g_i \bar{c}_{ix}, \nonumber \\ k_{2,T}= & \sum _{i=0}^{18} g_i \bar{c}_{iy}, \nonumber \\ k_{3,T}= & \sum _{i=0}^{18} g_i \bar{c}_{iz}. \end{aligned}$$

The post-collision distribution functions are calculated through

33 $$\begin{aligned} |g_i^{\star }\rangle = \textbf{T}^{-1} |k_{i, T}^{\star } \rangle , \end{aligned}$$

and streamed by Eq. (28). Similar to the works  [41, 42], the two-step approach is employed to calculated the post-collision distribution functions from central moments, and the interested reader can find the resultant expression in Appendix. Eventually, the macroscopic temperature is computed by the Eq. (15).

For the FVM solver using *code_saturne*, identical boundary type is applied to the velocity components due to the coupled treatment of velocity solver. Besides, the constant and adiabatic thermal boundary conditions are employed in the temperature field [27]. On the LBM side solved by LUMA, the boundary conditions for the velocity and temperature fields are imposed as follows: the no-slip and adiabatic wall conditions via the bounce-back scheme [34, 43], which defines as $f_{\bar{i}}(\boldsymbol{x}, t+\Delta t)= f_i^{\star }(\boldsymbol{x}, t)$ and $g_{\bar{i}}(\boldsymbol{x}, t+\Delta t)= g_i^{\star }(\boldsymbol{x}, t)$ where $\bar{i}$ is the reflected direction of *i*; velocity inlets using the regularised boundary method [33], i.e., $f_i(\boldsymbol{x}, t+\Delta t)=f_i^{eq}(\boldsymbol{x}, t+\Delta t)+(w_i \boldsymbol{Q:\Pi }^{(1)})/2c_s^4$, and constant temperature following Ref. [44].

### Coupling process between FVM and LBM

**Fig. 1:**
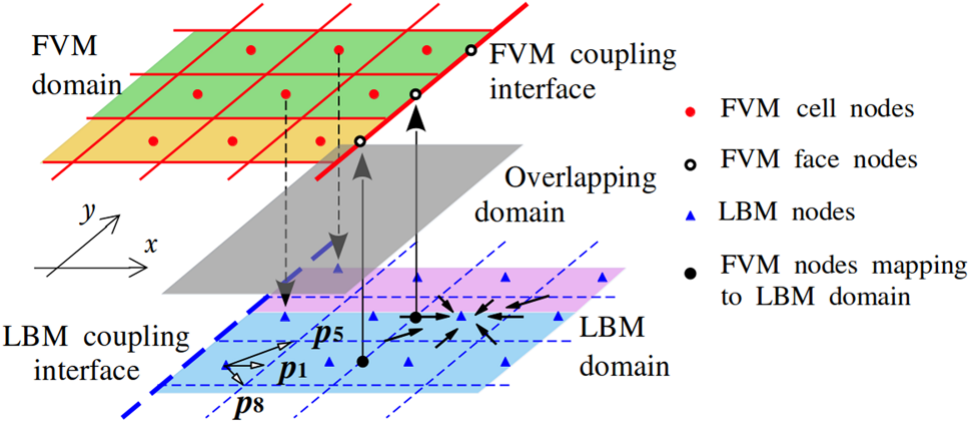
Spatial coupling between the FVM and LBM. The yellow, green, blue and purple regions represent MPI ranks

In our framework, the computational domain is partitioned into subregions, independently solved by the FVM and LBM. As shown in Fig. 1, both solvers operate on grids with identical spatial resolution: the FVM computes the solution over the solid red grid region, while the LBM operates within the blue dashed lattice region. A grey overlapping buffer zone, where both methods coexist, facilitates a smooth and stable coupling interface. Coupling is enforced at designated boundaries by exchanging macroscopic physical quantities such as velocity $\boldsymbol{u}$ and temperature *T*. The process begins by establishing a geometric correspondence between interface points, matching spatial coordinates across the FVM and LBM domains. Once identified, a two-way data exchange is performed, with macroscopic variables transferred to the corresponding solver interfaces, as indicated by the black arrows in Fig. 1. Dirichlet boundary conditions are imposed on both sides to ensure continuity. For parallel execution, the domain is further divided among multiple MPI ranks, shown in yellow, green, blue, and purple. The entire coupling procedure—including dynamic mapping generation, data exchange, and synchronisation at each coupling step—is managed by the PLE library [27], ensuring efficient and scalable communication between the FVM and LBM solvers.

The identical grid density across solvers enables a direct one-to-one mapping between FVM cell centres and LBM lattice nodes at the coupling interface. However, due to the staggered arrangement of the grids, FVM interface points correspond to interstitial positions relative to the LBM lattice. To ensure accurate LBM-to-FVM information transfer, a second-order Taylor-series-based interpolation scheme is employed, reconstructing macroscopic variables at the FVM coupling interface using data from neighbouring LBM nodes. The required spatial derivatives for this interpolation are computed using the gradient formulation [45]

34 $$\begin{aligned} & \boldsymbol{\nabla }\phi \left( \boldsymbol{x} \right) = \frac{1}{c_s^2} \sum _i^{18} w_i \boldsymbol{c}_i \phi \left( \boldsymbol{x} +\boldsymbol{c}_i \right) , \end{aligned}$$

where $\phi$ is a certain physical quantity to be interpolated. $w_i$ are the weights of the selected lattice discretisation, which are $w_0=1/3$, $w_{1-6}=1/18$ and $w_{7-18}=1/36$.

Macroscopic variables obtained from Eqs. (13)–(15) are directly transferred from the LBM to FVM after a suitable unit conversion. In contrast, when transferring velocity and temperature from FVM to LBM, these macroscopic quantities must be transformed into the corresponding unknown distribution functions (namely $p_1$, $p_5$ and $p_8$ as shown in Fig. 1). Here, we reconstruct these by implementing the regularized boundary approach [33] to determine the distribution functions of the velocity field, which are expressed as

35 $$\begin{aligned} & f_i = f_i^{eq} + \frac{w_i}{2c_s^4} \boldsymbol{Q}: \boldsymbol{\Pi }^{(1)}, \end{aligned}$$

where $\displaystyle \boldsymbol{Q} = \boldsymbol{c}_i \boldsymbol{c}_i - c_s^2\textbf{I}$ and $\displaystyle \boldsymbol{\Pi }^{(1)} = \sum _i \boldsymbol{c}_i \boldsymbol{c}_i \left( f_i -f_i^{eq} \right)$. At the coupling interface, the unknown distribution functions for the temperature field are determined using the extended equilibrium distribution functions, i.e. $\displaystyle g_i = g_i^{eq}$ as specified in Eq. (12).

**Fig. 2:**
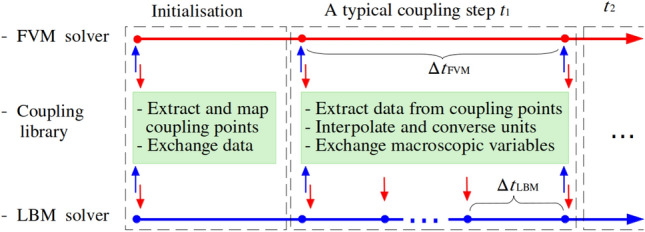
Temporal coupling process between FVM and LBM

Beyond spatial coupling, careful treatment of the temporal coupling scheme is equally critical. Standard stability requirements must be satisfied by both solvers: a Courant number of unity for FVM and a lattice velocity much smaller than the speed of sound for LBM. Furthermore, while FVM simulates incompressible flows using an implicit time integration scheme, LBM adopts an explicit one, leading to inherently larger time steps for FVM compared to LBM. This temporal mismatch requires a specialised coupling strategy, illustrated in Fig. 2. The coupling process unfolds in two stages: first, interface points are identified across FVM and LBM domains during the initialisation phase, establishing a mapping relationship and performing an initial data exchange. Then, during the simulation, each FVM time step encompasses multiple LBM sub-iterations ($\Delta t_{\textrm{FVM}} = n\Delta t_{\textrm{LBM}}$, with $n \ge 1$). Throughout these LBM sub-iterations, the coupling interface information remains fixed, preserving the transformation data from the previous FVM-LBM exchange.

In the present work, we implement a coupled computational framework combining the FVM through *code_saturne* [25] and the LBM via LUMA [26], integrated using the PLE coupling library [46]. On the LBM side, the LUMA solver employs a two-array streaming algorithm to advance the distribution functions. To show the coupling procedure, consider a minimal configuration with two MPI ranks, each assigned to each solver. From an algorithmic perspective, each coupling time step executes the following sequence of operations:

1. update macroscopic variables for both LBM by Eqs. (13),  (14) and (15) and FVM;
2. compute the gradient fields on the LBM side using neighbouring points as indicated by the arrows in Fig. 1 through Eq. (34).
3. predict macroscopic variables at coupling points using the second-order Taylor series on the LBM side;
4. exchange macroscopic variables between solvers via the PLE coupling library;
5. reconstruct unknown populations through the regularised scheme and the equilibrium distribution for velocity and temperature fields on the LBM coupling interface by Eqs. (35) and (12), respectively;
6. advance one FVM and *n* LBM time steps, and return to action 1.

Additionally, Algorithm 1 presents a pseudo code for the spatial FVM-LBM coupling interface treatment, where $\mathrm {F_{FVM}}$ and $\mathrm {F_{LBM}}$ denote the coupling interface points on the FVM and LBM sides, while $\Omega _{\textrm{FVM}}$ and $\Omega _{\textrm{LBM}}$ represent the coupling interface points mapping to FVM and LBM subdomains.

**Algorithm 1:**
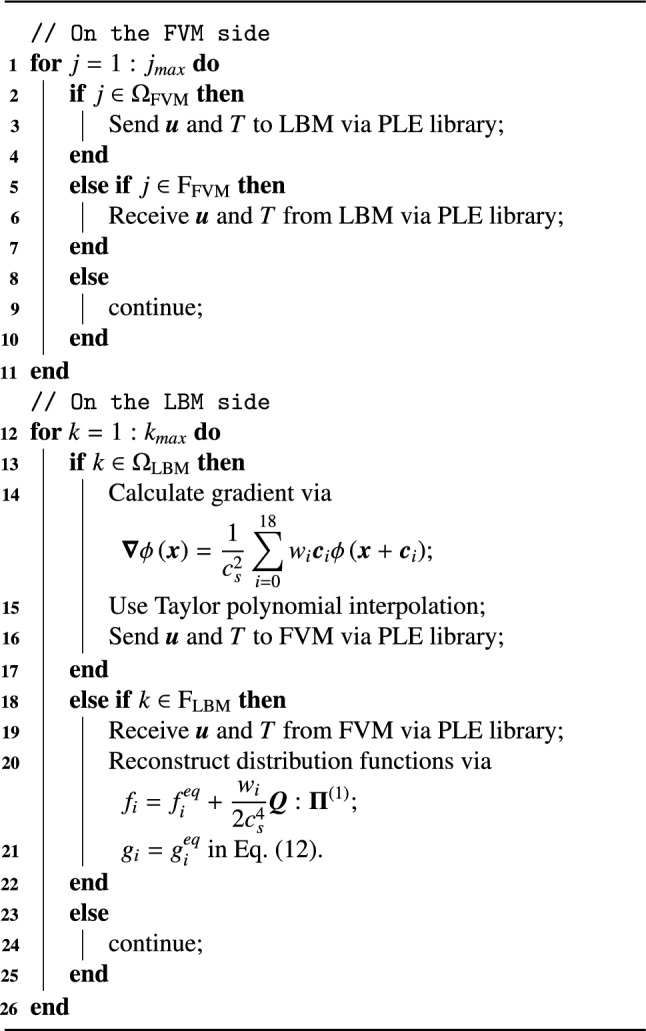
Pseudo code for the FVM-LBM coupling interface treatment.

## Results

To assess the performance, robustness, and versatility of the proposed coupling framework, we validate it against a suite of seven benchmark problems designed to progressively challenge different physical and numerical aspects of the method.

- One-dimensional heat conduction: tests the performance of the coupling framework in purely diffusive regimes, including the sensitive analysis of grid resolution, overlapping size, number of LBM sub-iterations, and varying material properties across the FVM-LBM boundary.
- Two-dimensional natural convection in a cavity: examines the ability to resolve buoyancy-driven flows at Rayleigh numbers of $10^5$ and $10^6$, with particular attention to thermal stability and mass conservation across the coupling interfaces.
- Two-dimensional natural convection in a side-open cavity with porous media: evaluates heat and momentum transport in complex solid–fluid configurations, a common feature in energy systems such as solar receivers and cooling devices.
- Two-dimensional normal plate velocity: introduces forced convection with vertical inflow and a moving upper boundary. This setup not only provides an analytical solution for validation but also lays the groundwork for future extensions involving fully moving geometries.
- Two-dimensional thermal lid-driven cavity: combines shear-driven flow and thermal gradients, testing the framework’s capacity to capture secondary recirculation effects and mixed convection regimes.
- Three-dimensional natural convection in a cavity: extends the two-dimensional setup to full 3D and evaluates the framework’s capability to handle large-scale, fully coupled simulations in realistic geometries.
- Rayleigh-Bénard convection with a melting boundary: demonstrates the framework’s applicability to evolving boundary problems and multiphase thermofluid dynamics, employing the hybrid strengths of FVM and LBM for tracking moving solid–liquid interfaces.

Together, these cases provide a comprehensive validation across a broad spectrum of physical regimes and modelling challenges, supporting the applicability of the framework to industrial and scientific problems.

### One-dimensional heat conduction

**Fig. 3:**
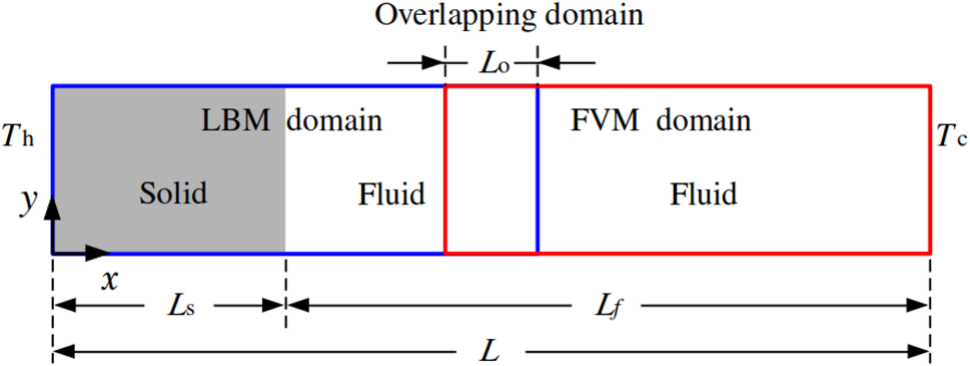
One-dimensional heat conduction: sketch of the problem setup

Figure 3 presents a heat conduction problem designed to evaluate the numerical accuracy of the coupling model. The computational domain of length $L = 1$ is divided into LBM (blue) and FVM (red) subregions, occupying 0.5*L* and 0.54*L* in the *x*-direction, respectively. A solid region of length $L_s = 0.2L$ is placed to the left of the LBM domain, while the remaining portion is occupied by a fluid. An overlapping region of length $L_o = 0.04L$ is shared between the two solvers to ensure a smooth coupling interface. Since the temperature distribution is independent of the *y*-direction, only five grid points are used in that direction.

Constant high ($T_h$) and low ($T_c$) temperatures are imposed at the left and right boundaries, respectively, while periodic boundary conditions are applied elsewhere. The initial temperature is set to $T_0 = (T_h + T_c)/2$. To investigate the coupling performance across varying material properties, the thermal conductivity ratio between solid and fluid, defined as $\kappa = \lambda _s / \lambda _f$, is varied as $\kappa = 0.1$, 1, 10, and 100.

According to Eq. (3), the steady-state solution for heat conduction without a source term satisfies:

36 $$\begin{aligned} \boldsymbol{\nabla } \cdot \left[ \alpha _{(s,f)} \boldsymbol{\nabla } T \right] = 0, \end{aligned}$$

where $\alpha _s$ and $\alpha _f$ denote the thermal diffusivities of the solid and fluid regions, respectively. For convenience, we introduce the dimensionless temperature $\theta$ as

37 $$\begin{aligned} \theta = \frac{T-T_c}{T_h-T_c}. \end{aligned}$$

Eq. (36) admits analytical solution in the form

$$ \theta (x) = {\left\{ \begin{array}{ll} a_s x + 1, & \text {for} \quad 0 \le x \le L_s,\\[8pt] a_f x + b_f, & \text {for} \quad L_s < x \le L, \end{array}\right. } $$

where

$$ a_s = -\frac{1}{L_s + (L - L_s)\kappa }, \quad a_f = \kappa a_s, \quad b_f = 1 + a_s L_s (1 - \kappa ). $$

For a more quantitative assessment of the accuracy of the proposed methodology, the $l^2$-norm of the relative error between numerical ($\boldsymbol{\sigma }_{\textrm{cp}}$) and analytical ($\boldsymbol{\sigma }_{\textrm{an}}$) solutions is computed as

38 $$\begin{aligned} \epsilon = \frac{| \boldsymbol{\sigma }_{\textrm{an}} - \boldsymbol{\sigma }_{\textrm{cp}} |}{| \boldsymbol{\sigma }_{\textrm{an}} |} \times 100\% \end{aligned}$$

**Fig. 4:**
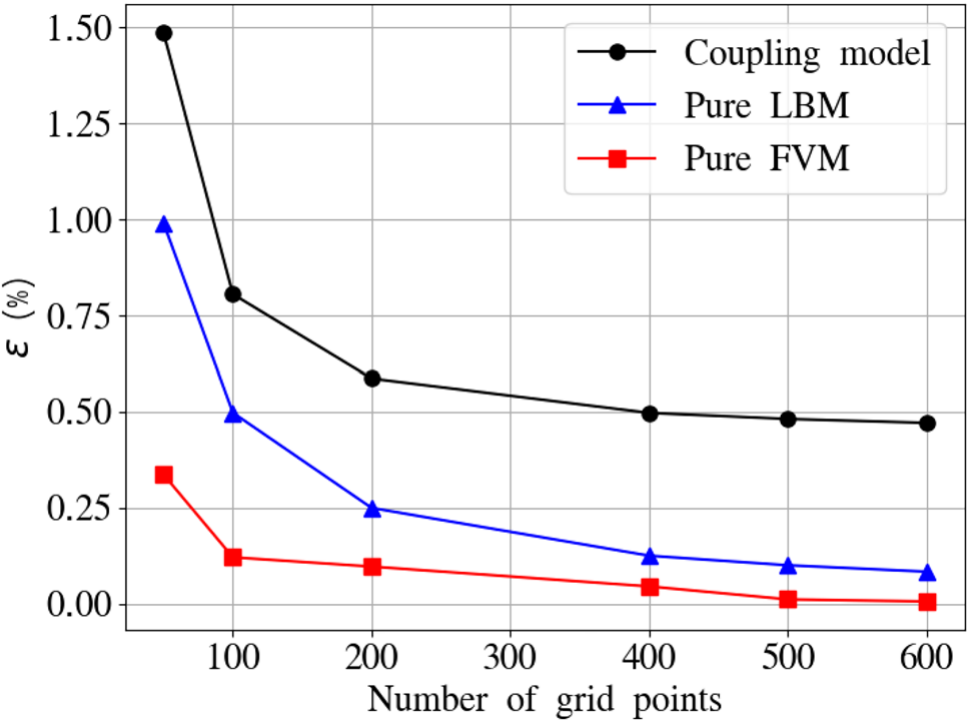
One-dimensional heat conduction: grid independence study

Figure 4 illustrates the variation of the relative error at $\kappa =1$ as a function of grid resolution ($1/50,\,1/100,\,1/200,\,1/400,\,1/500$ and 1/600) for the pure FVM, pure LBM, and coupling models. The time steps are set to 0.002 for the LBM and 0.02 for the FVM in all simulations, with ten LBM sub-iterations performed within each FVM time step in the coupling framework. The relative error decreases monotonically with increasing grid resolution for all the considered models, with the FVM showing superior performance. In the coupled simulation, the relative error decreases to $0.48~\%$ at a resolution of 1/500, which is therefore adopted for the subsequent studies.

The influence of the overlapping domain is further investigated using $L_o=4,\,8,\,12,\,16,\,20$ and 30 grid points. Table 1 shows the relative error decreases continuously as the overlapping size increases. The behaviour arises because the equilibrium distribution functions are employed as the reconstruction operator on the LBM coupling interface, and they depend on the local velocity and temperature fields.

**Table 1:** One-dimensional heat conduction: percentage relative discrepancy for different overlapping size

$L_o$ (grid points)	4	8	12	16	20	30
$\epsilon \, [\%]$	2.23	1.11	0.76	0.58	0.48	0.34

Additionally, the study of number of LBM sub-iteration (*n*) in Table 2 shows that the relative error gradually decreases as *n* increases. For a fixed grid resolution, increasing *n* reduces the LBM time step, which in turn decreases the thermal diffusivity in lattice unit ($\alpha$) and increases the relaxation frequency, thereby further improving the numerical accuracy [34].

**Table 2:** One-dimensional heat conduction: percentage relative discrepancy for different LBM sub-iteration (*n*)

*n*	1	2	4	8	10	16	20
$\epsilon \, [\%]$	4.00	2.13	1.11	0.59	0.48	0.32	0.26

**Fig. 5:**
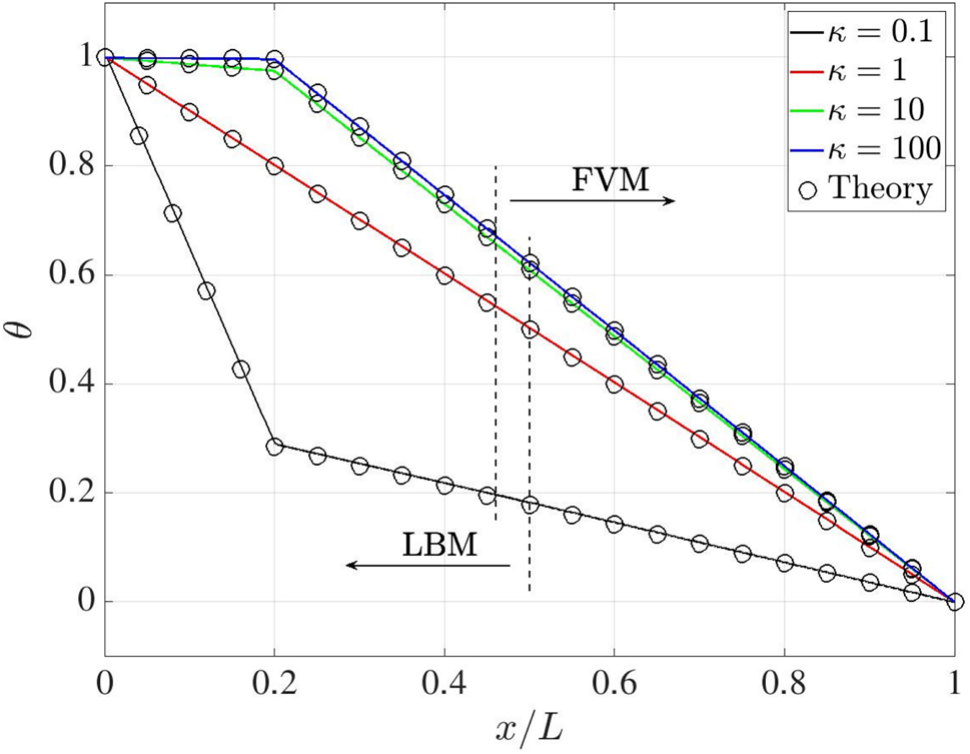
One-dimensional heat conduction: dimensionless temperature distribution for different values of $\kappa$, i.e. $\kappa = 0.1$ (black), 1 (red), 10 (green), and 100 (blue). Present results and analytical predictions are represented by lines and circles, respectively

Figure 5 compares the dimensionless temperature profiles obtained from the coupled model and the analytical solution for different values of $\kappa$. Our results show excellent agreement with the theoretical prediction across all cases. In the overlapping region, the FVM and LBM yield identical temperature fields, and a smooth transition is observed across the coupling interface.

To quantitatively assess the conjugated heat transfer performance, Table 3 reports the relative discrepancy ($\epsilon$) at $\kappa = 0.1$, 1, 10, and 100. The very slight mismatch confirms the high accuracy and robustness of our thermal coupling model.

**Table 3:** One-dimensional heat conduction: percentage relative discrepancy between present results and analytical predictions for different $\kappa$

$\kappa$	0.1	1	10	100
$\epsilon \, [\%]$	1.23	0.48	0.37	0.35

### Two-dimensional natural convection in a cavity

**Fig. 6:**
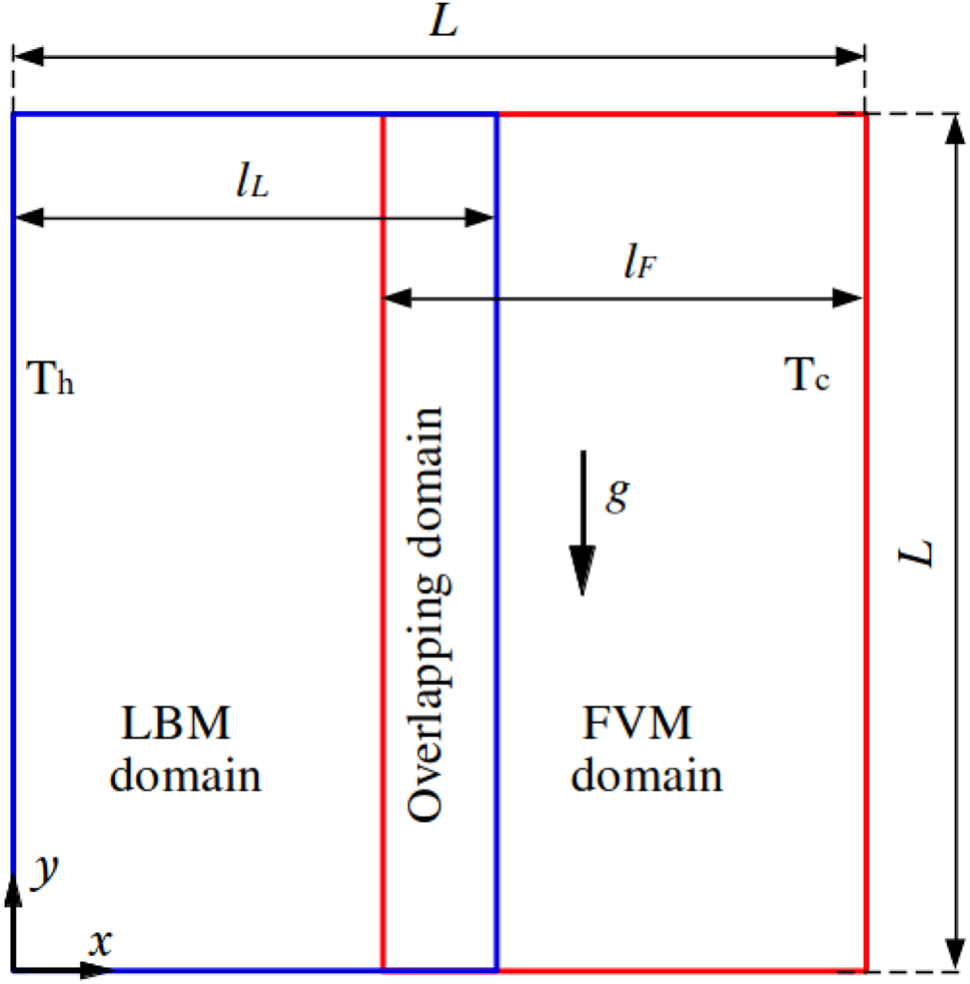
Two-dimensional natural convection in a cavity: sketch of the problem setup

The present coupling framework is further validated against the two-dimensional natural convection problem. Figure 6 sketches the computational domain of size $L\times L$, where the blue and red sub-regions of size $0.5L\times L$ and $0.54L\times L$ are simulated by the LBM and FVM, respectively, with an overlapping domain ($0.04L\times L$). The entire domain adopts the same uniform resolution, i.e., 1/500.

Constant high ($\theta _h=1$) and low temperatures ($\theta _c=0$) are enforced on the left and right borders, while the top and bottom ones use the adiabatic condition. No-slip boundary conditions are enforced at each side. The fluid density is set to $\rho =1.0$ with the kinetic viscosity $\nu = 2.0\times 10^{-4}$. At the beginning of the simulation, a quiescent fluid of temperature $\theta _0 = 0.5$ is considered. For each FVM time step ($\Delta t = 0.02$), the LBM performs 10 sub-iterations with time step of 0.002. Gravity is enforced along the *y* direction and set to $g=-9.806$. Values of the Rayleigh number of $10^5$ and $10^6$ are investigated in this study. The Prandtl number is set to Pr$=0.71$.

**Fig. 7:**
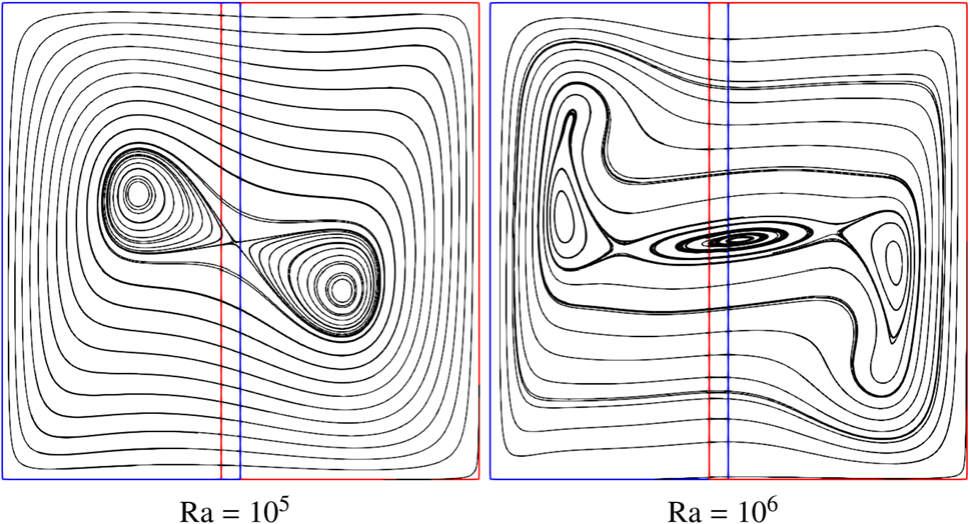
Two-dimensional natural convection in a cavity: streamline of the velocity field at Ra $= 10^5$ and $10^6$

Figure 7 shows the velocity field with streamlines at Ra $=10^5$ and $10^6$. A smooth flow field can be observed throughout the computational domain, especially near the coupling interfaces. The heated fluid ascends along the left wall due to the buoyancy force, impinges on the top face, travels toward the cold wall, and finally sinks near the cooling wall. Meanwhile, a reverse flow can be found from the cooling to the heating surface, leading to a steady clockwise rotational flow in the cavity. Finally, two independent vortices are generated in the central domain at Ra $=10^5$, while two vortices move toward the heating and cooling walls and the third vortex occurs in the centre of domain at Ra $=10^6$. Using traditional pure FVM method, Chen et al. [47] and Luan et al. [21] numerically investigated two-dimensional natural convection at Ra $=10^5$ and $10^6$ leveraging commercial software FLUENT. These findings are in excellent agreement with previous efforts [21, 47].

**Fig. 8:**
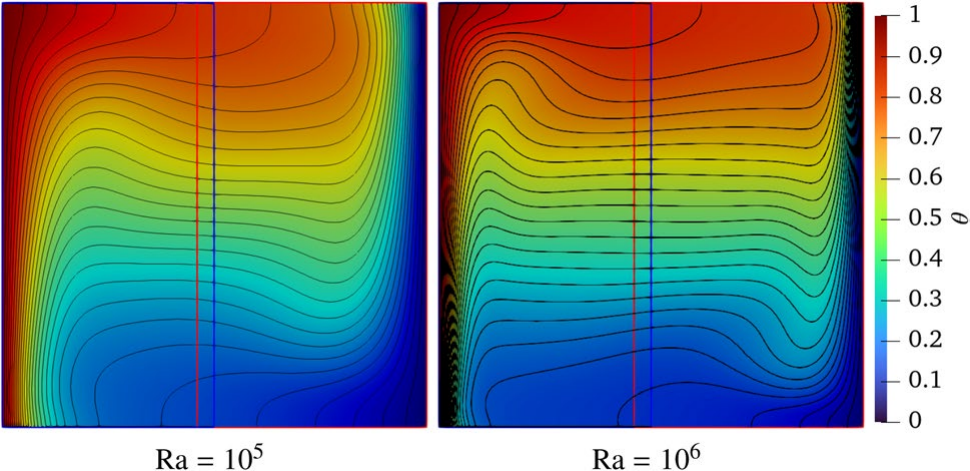
Two-dimensional natural convection in a cavity: temperature field with isotherms at Ra $=10^5$ and $=10^6$

Figure 8 displays the temperature field at Ra $=10^5$ and $10^6$, demonstrating that the isotherms smoothly transition the coupling interfaces. Their distributions match well those shown in other studies [21, 47]. Notably, at Ra $=10^6$, Li et al. [10] observed visible discontinuities at the FVM–LBM interfaces, which can be attributed to inaccuracies introduced by the non-equilibrium extrapolation method used for reconstructing flow and thermal fields (see Figure 16 in Ref. [10]). In contrast, our coupling approach eliminates these discontinuities, ensuring smooth temperature profiles across the interface.

**Fig. 9:**
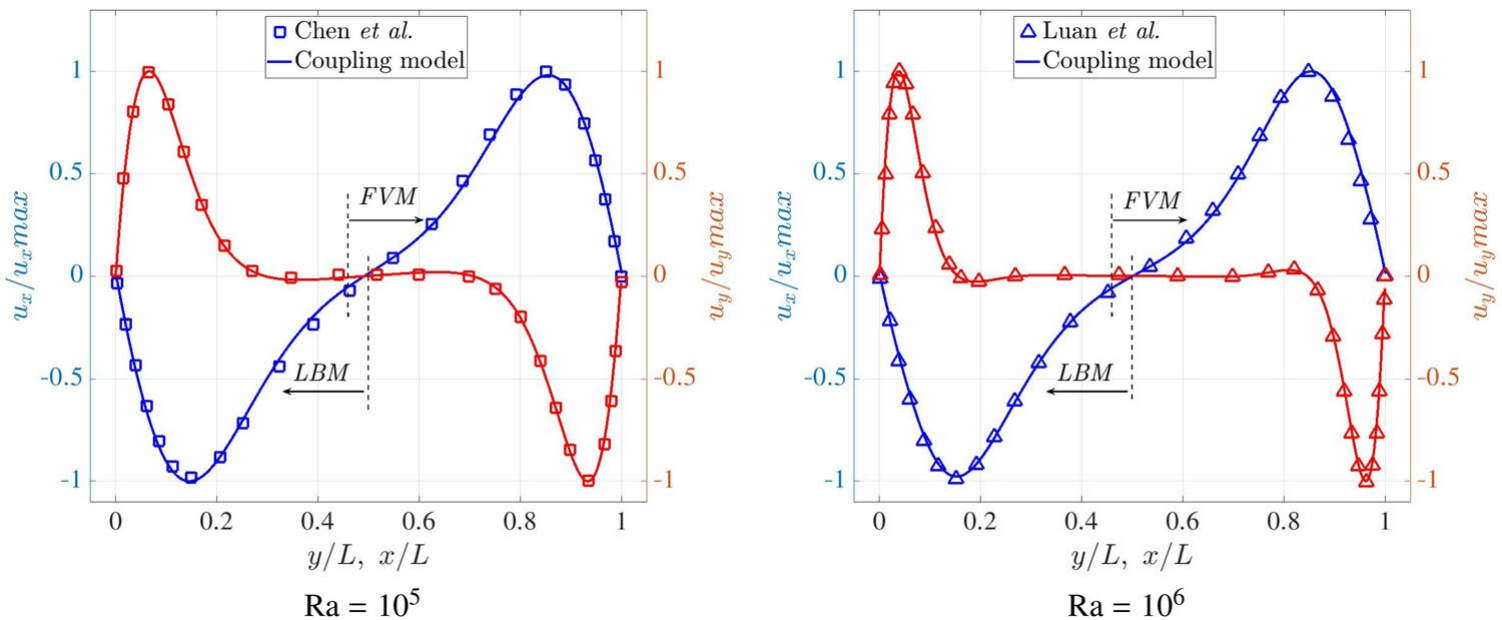
Two-dimensional natural convection in a cavity: velocity distributions along the midlines $x/L=0.5$ and $y/L=0.5$ at Ra $=10^5$ and $10^6$

To quantify the accuracy of coupling model, Fig. 9 shows the distributions of dimensionless velocity components along $y/L=0.5$ and $x/L=0.5$ at Ra $=10^5$ and $10^6$. The velocity component ($u_x$) is normalised by the maximum value ($u_{x,max}$) on the midline $x/L=0.5$, and $u_y$ is normalised by the maximum value ($u_{y,max}$) on the midline $y/L=0.5$. Note that the midline of $y/L=0.5$ crosses the LBM, FVM domains and the coupling interfaces while the whole line of $x/L=0.5$ is in the FVM domain. Similarly, the velocity profiles obtained from the coupling framework are in excellent agreement with the references [21, 47].

**Fig. 10:**
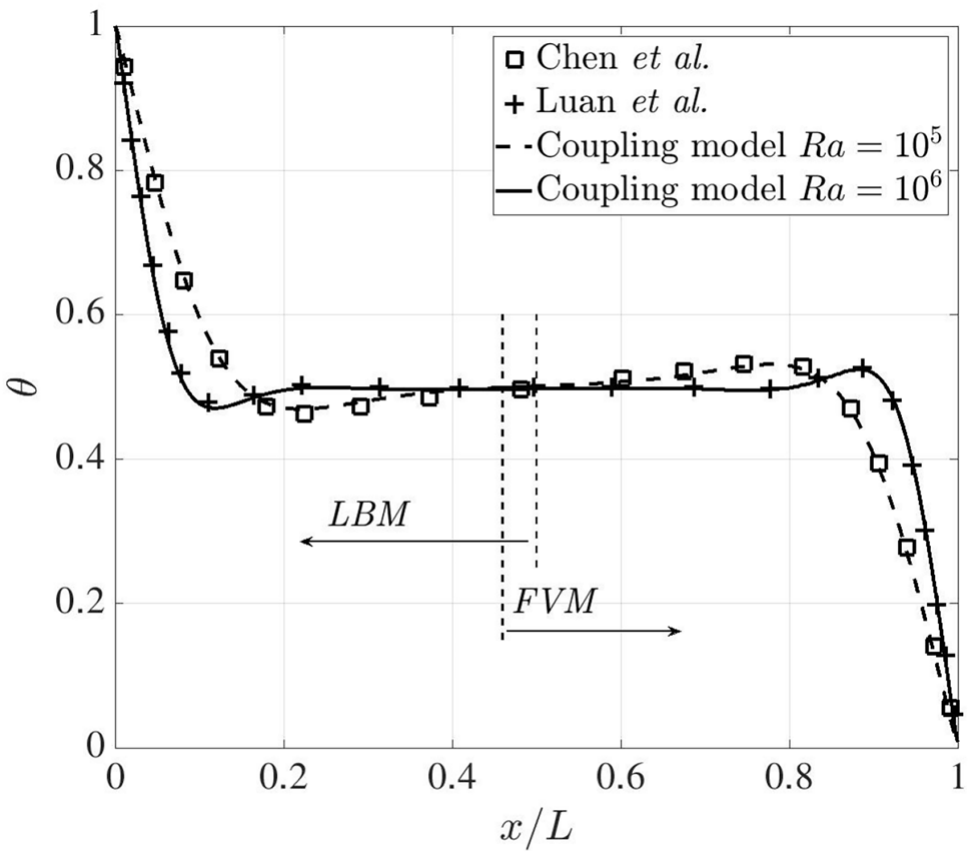
Two-dimensional natural convection in a cavity: temperature distribution along the midline $y/L=0.5$ at Ra $=10^5$ and $10^6$

In Fig. 10, the dimensionless temperature distributions are quantitatively compared with the references [21, 47] on the midline of $y/L=0.5$. The temperature profiles change rapidly close to the heating and cooling surfaces, while they are relatively flat towards the centre of computational domain. They remain in excellent agreement with the reference data throughout. In addition, the temperature profiles are entirely continuous across the coupling interfaces, demonstrating the robustness of the coupling implementation.

To quantitatively assess heat transfer performance, Davis [48] employed second-order finite difference method to study two-dimensional natural convection and analysed the Nusselt numbers. The local ($\textrm{Nu}$) and averaged ($\overline{\textrm{Nu}}$) Nusselt numbers are calculated on the heating wall and they are defined as

39 $$\begin{aligned} & \textrm{Nu} = - {L\over \Delta T} \left( {\partial T \over \partial x}\right) , \end{aligned}$$

40 $$\begin{aligned} & \overline{\textrm{Nu}} = \int _0^L \textrm{Nu} \, dy. \end{aligned}$$

Tables 4 and 5 list the averaged, maximum and minimum values of the Nusselt number and the corresponding location on the heating surface at Ra $=10^5$ and $10^6$. The five parameters obtained from the coupling model match well with those in Refs. [21, 48].

**Table 4:** Comparison of Nusselt number on the heating wall at Ra $=10^5$

	Present	Davis [48]	Luan et al. [21]
$\overline{\textrm{Nu}}$	4.505	4.510	4.507
$Nu_{max}$	7.742	7.761	7.738
$(y/L)_{max}$	0.082	0.085	0.083
$Nu_{min}$	0.700	0.736	0.746
$(y/L)_{min}$	1.000	1.000	0.997

**Table 5:** Comparison of Nusselt number on the heating wall at Ra $=10^6$

	Present	Davis [48]	Luan et al. [21]
$\overline{\textrm{Nu}}$	8.803	8.928	8.807
$Nu_{max}$	17.686	18.076	17.71
$(y/L)_{max}$	0.0375	0.0456	0.0375
$Nu_{min}$	0.996	1.005	0.978
$(y/L)_{min}$	1.000	1.000	0.997

**Fig. 11:**
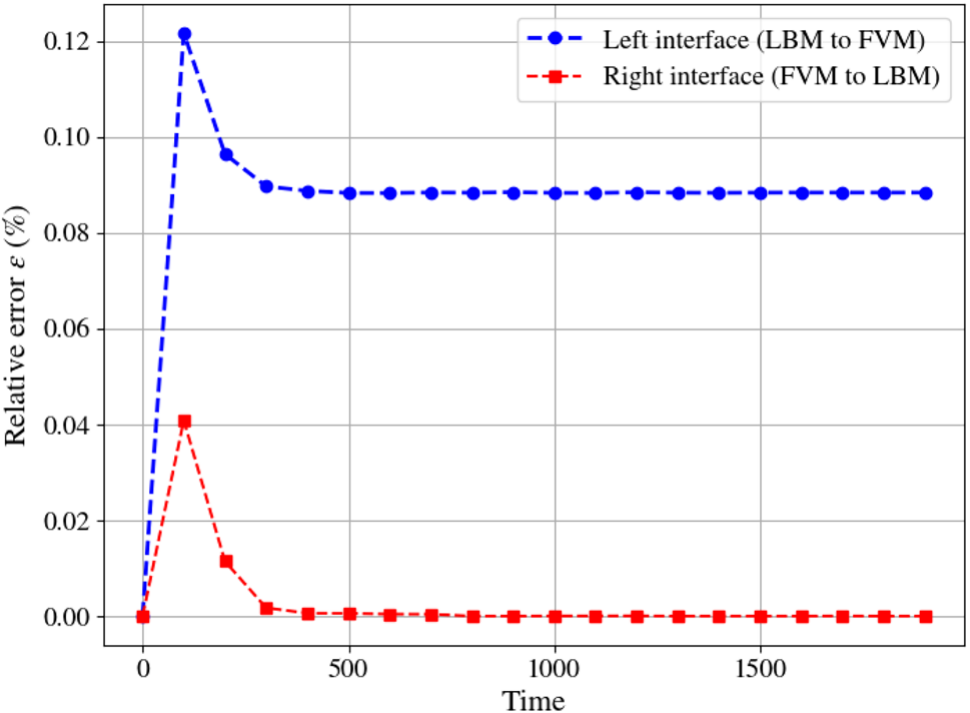
Two-dimensional natural convection in a cavity: change of relative error of mass flux with time at the coupling interfaces

At Ra=$10^6$, mass conservation is further assessed at the coupling interfaces by monitoring the relative error of the mass flux ($\rho u_x$, see Eq. 38) as a function of time, as shown in Fig. 11. Referring to Fig. 6, the left coupling interface corresponds to data transfer from LBM to FVM, while the right interface represents transfer from FVM to LBM. The results show that the error converges to approximately $0.09~\%$ at the left interface, while it becomes negligible at the right interface. The slightly higher error at the left interface is attributed to the fact that LBM data are stored at face locations, whereas the monitored quantities are evaluated at cell centres.

**Fig. 12:**
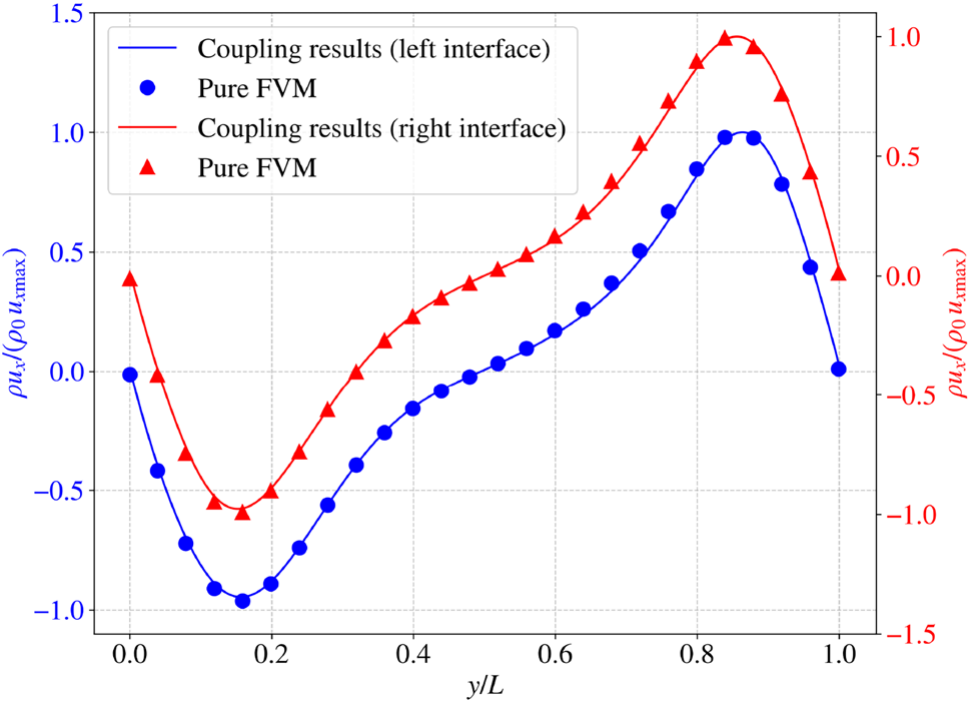
Two-dimensional natural convection in a cavity: comparison of normalized mass flux at the left and right interfaces between the coupling and pure FVM models

After convergence is reached, the dimensionless mass flux is compared between the coupling and pure FVM models at the left and right interfaces, with the pure FVM data extracted at the corresponding interface locations. The results indicate good agreement between the two methods, with relative errors ($\varepsilon$) of only $2.03\%$ at both interfaces, demonstrating the accuracy of mass conservation in the coupled framework (Fig. 12).

Furthermore, the relative error of mass flux between the coupling and pure FVM models is examined for different numbers of LBM sub-iterations per FVM time step, with the results summarized in Table 6. For all five cases, the grid resolution over the entire domain is fixed at 1/500, and the FVM time step is maintained at 0.02. The results show that the errors decrease monotonically with increasing *n* at both interfaces, and similar error levels are observed at left and right interface of each case. This trend can be attributed to the weak compressibility inherent in the LBM, as increasing *n* effectively reduces the LBM time step and lattice velocity which is directly related to compressibility-induced errors.

**Table 6:** Two-dimensional natural convection in a cavity: relative error of mass flux between the coupling and pure FVM models

$\Delta t_{FVM}/\Delta t_{LBM}$ (*n*)	Left interface	Right interface
4	9.25%	8.98%
8	2.82%	2.79%
10	2.03%	2.03%
16	1.19%	1.20%
20	1.02%	1.03%

**Fig. 13:**
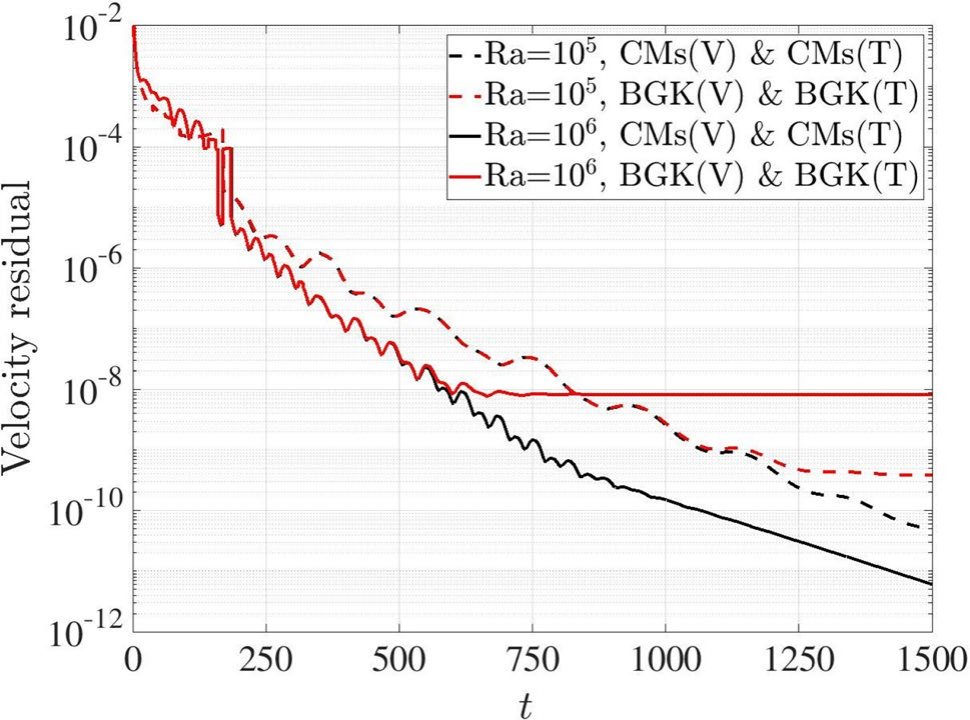
Two-dimensional natural convection in a cavity: variations of velocity residuals with time steps for BGK- and CMs-LBM coupling schemes at Ra $=10^5$ and $10^6$

The benefit of introducing the CMs-LBM scheme is evaluated with reference to the steady-state convergence for both cases Ra $=10^5$ and $10^6$. The velocity and temperature residuals are defined in Eqs. (41) and (42) [47] as

41 $$\begin{aligned} \mathrm {Velocity\ residual} = \frac{ \left\| \textbf{u}(t+\delta t) - \textbf{u}(t) \right\| _2 }{ \left\| \textbf{u}(t+\delta t) \right\| _2 }, \end{aligned}$$

42 $$\begin{aligned} \mathrm {Temperature\ residual} = \sum _{x,y} \left| \frac{ T(x,y,t+\delta t) - T(x,y,t) }{ T(x,y,t+\delta t) } \right| , \end{aligned}$$

where $\Vert \cdot \Vert _2$ is the discrete $l^2$ norm over the domain. Figure 13 compares the velocity residual variations with dimensionless physical time between the BGK and CMs coupling schemes at Ra $=10^5$ and $10^6$. At Ra $=10^5$, the same variation is observed at initial stage ($t < 1100$). Then the residual remains at about $10^{-9}$ for BGK scheme while the CMs scheme captures further decreases. A similar phenomenon is observed at Ra $=10^6$. The velocity residual starts to diverge at about $t=600$, after which the BGK scheme remains around $10^{-8}$, whereas the CM schemes exhibit a continuous decrease.

**Fig. 14:**
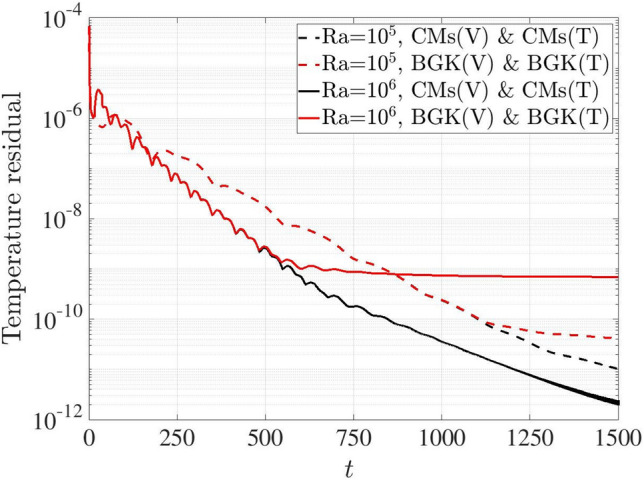
Two-dimensional natural convection in a cavity: variation of temperature residuals with time steps for BGK- and CMs-LBM coupling schemes at Ra $=10^5$ and $10^6$

Figure 14 depicts the time history of temperature residuals based on thermal BGK and CMs coupling schemes. Using the BGK scheme, the coupling model converges to approximately $10^{-9}$ and $10^{-10}$ at Ra $=10^5$ and $10^6$, respectively, while the temperature residuals continue to decrease for the CMs scheme.

According to the analysis from the Figs. 13 and 14, the residuals from CMs scheme are lower than those of the BGK scheme in both flow and temperature fields, indicating the superior numerical convergence and stability of the CMs scheme in coupling model.

**Fig. 15:**
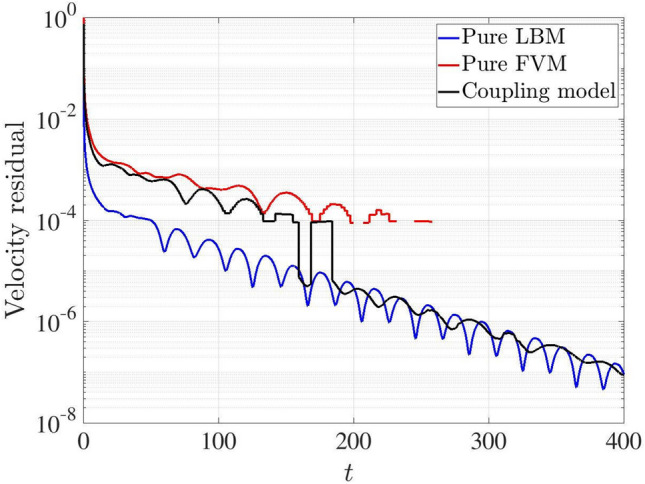
Two-dimensional natural convection in a cavity: variations of velocity residuals with time for the pure FVM, pure LBM and coupling models at Ra $=10^6$

At Ra$=10^6$, Fig. 15 shows the variation of the velocity residual with dimensionless physical time for the pure FVM, pure LBM and the coupled models. The discontinuity in the pure FVM result and the abrupt jump in the coupling model indicate that the velocity residual reaches zero for the FVM calculation. The excellent convergence performance of pure LBM ascribes to its smaller time step, i.e., $\Delta t_{LBM}=0.002$ and $\Delta t_{FVM}=0.02$. When *t* is less than approximately 160, the residual of coupling model is comparable to that of the pure FVM, but remains higher than that of pure LBM model. Once the residual from the pure FVM calculation drops to zero, the residual of coupled model approaches that of pure LBM. These observations demonstrate that the convergence behaviour of the coupling model lies between those of the pure FVM and LBM methods in the flow field.

**Fig. 16:**
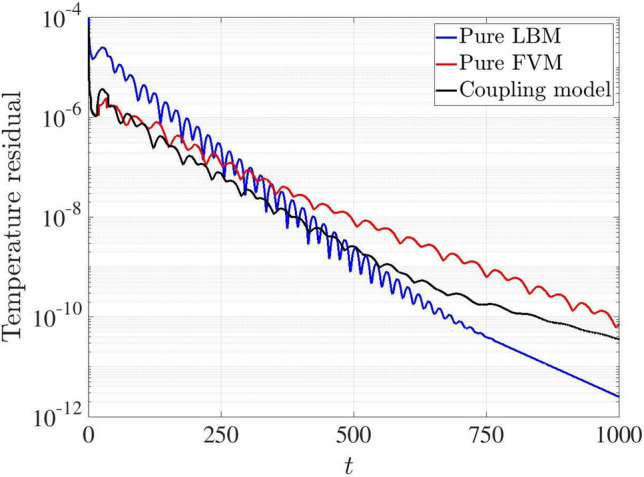
Two-dimensional natural convection in a cavity: variations of temperature residuals with time for the pure FVM, pure LBM and coupling models at Ra $=10^6$

The comparison of the temperature residual is illustrated in Fig. 16 for the pure FVM, pure LBM and the coupling models at Ra$=10^6$. The results indicate that the coupling model exhibits a faster initial convergence speed than both pure FVM and pure LBM models. Subsequently, the residual of the coupling model falls between those of the pure FVM and pure LBM simulations, demonstrating that its convergence performance lies between the pure FVM and pure LBM approaches.

### Two-dimensional natural convection in a side open cavity with porous media

Natural convection in a side open cavi ty with porous media has ubiquitous engineering applications, such as solar receivers, ventilation, cooling of electronic [49], which involves complex flow and solid–fluid heat transfer processes. Referring to the two-dimensional study in Ref. [50], the proposed coupling model is validated through the thermo-hydraulic effects of discrete solid blocks on the natural convection inside a fluid-filled, horizontally heated cavity with the opening to one side.

**Fig. 17:**
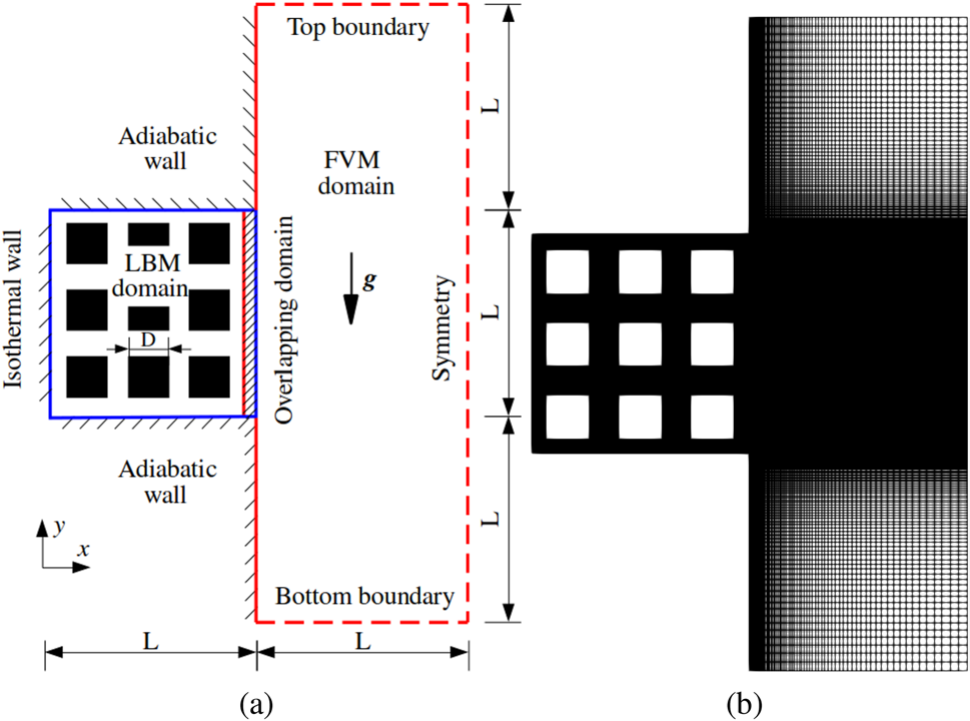
Two-dimensional natural convection in a side open cavity with porous media: geometrical configuration **a** and grid points distribution **b**

The configuration of entire computational domain is shown in Fig. 17a. The square cavity ($L\times L$) is simulated by LBM while the thermal reservoir in red domain is solved by FVM ($L\times 3L$), with the shadow overlapping domain ($0.04L\times L$) solved by both methods. There are nine conducting, disconnected and uniformly distributed square blocks and the side length is set to $D=0.2L$. Similarly to Ref. [50], the left boundary of the cavity is defined as a heating wall ($\theta _h=1.0$) while the remaining boundaries adopt an adiabatic boundary condition. In the thermal reservoir, the top and bottom borders enforce a constant lower temperature ($\theta _c=0.0$) with the zero-gradient velocity boundary while the right boundary is set to symmetry. Figure 17b illustrates the grid points distribution over the entire computational domain. The LBM portion is decomposed into uniform lattice points, with 500 points for the length of *L*. Each block is captured by $100\times 100$ lattice points, so the porosity equals to $\phi =0.64$ in the cavity. A non-uniform grid distribution is applied to the FVM domain with the mesh size ranging from 0.002 to 0.02. The overlapping domain consists of $20\times 500$ grids along the *x* and *y* directions. The temperature field is initialised as $\theta _c=0.0$ everywhere. The Rayleigh number is set to $\displaystyle Ra = g \beta \Delta T L^3/(\nu \alpha _f)=10^5$, where the $\nu$ and $\alpha _f$ being thermal diffusivity and kinematic viscosity of the fluids. The Prandtl number ($Pr=\nu /\alpha _f$) is set to 1.0. Different ratios of solid–fluid thermal diffusivity ($\kappa =\alpha _s/\alpha _f$, with $\alpha _s$ being thermal diffusivity of solid) are investigated, *i.e*, $\kappa = 0.1$, 1.0, 10 and 100, respectively.

**Fig. 18:**
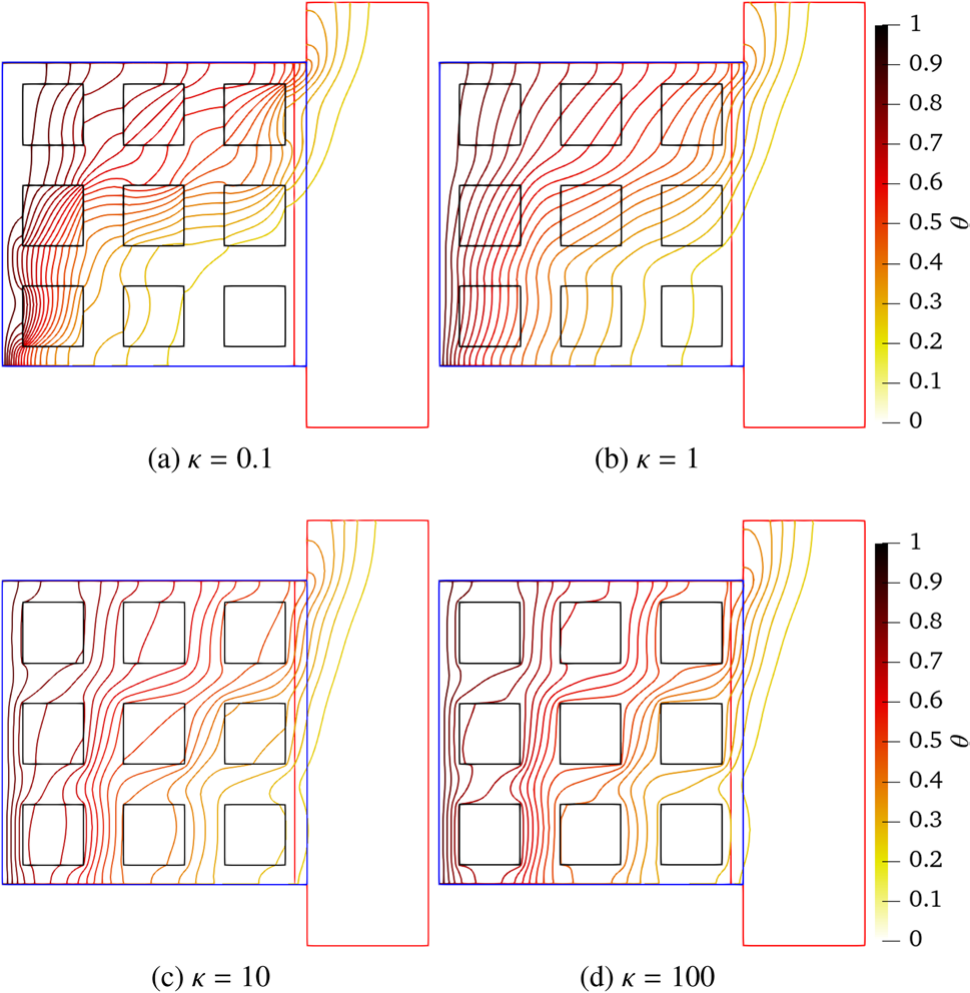
Two-dimensional natural convection in a side open cavity with porous media in a porous medium: isotherm distributions closed to the cavity at $\kappa =0.1$, 1, 10 and 100

Figure 18 exhibits the temperature field closed to the open cavity with the solid–fluid conductivity ratios ranging from 0.1, 1.0, 10 to 100. The temperature difference of two adjacent isotherms is set to 0.05 in Fig. 18. At $\kappa =0.1$, the dense isotherms occur in solid blocks due to the lower thermal diffusivity. As $\kappa$ increases, the effects of heat diffusion are gradually enhanced in blocks while the temperature distributions in the fluid are induced by the convection and diffusion effects. When $\kappa$ increases to 100, the isotherms concentrate on the fluid, meaning that the thermal resistance of the solid is smaller. Moreover, the isotherms smoothly cross the coupling interfaces which proves the correctness of coupling scheme.

**Fig. 19:**
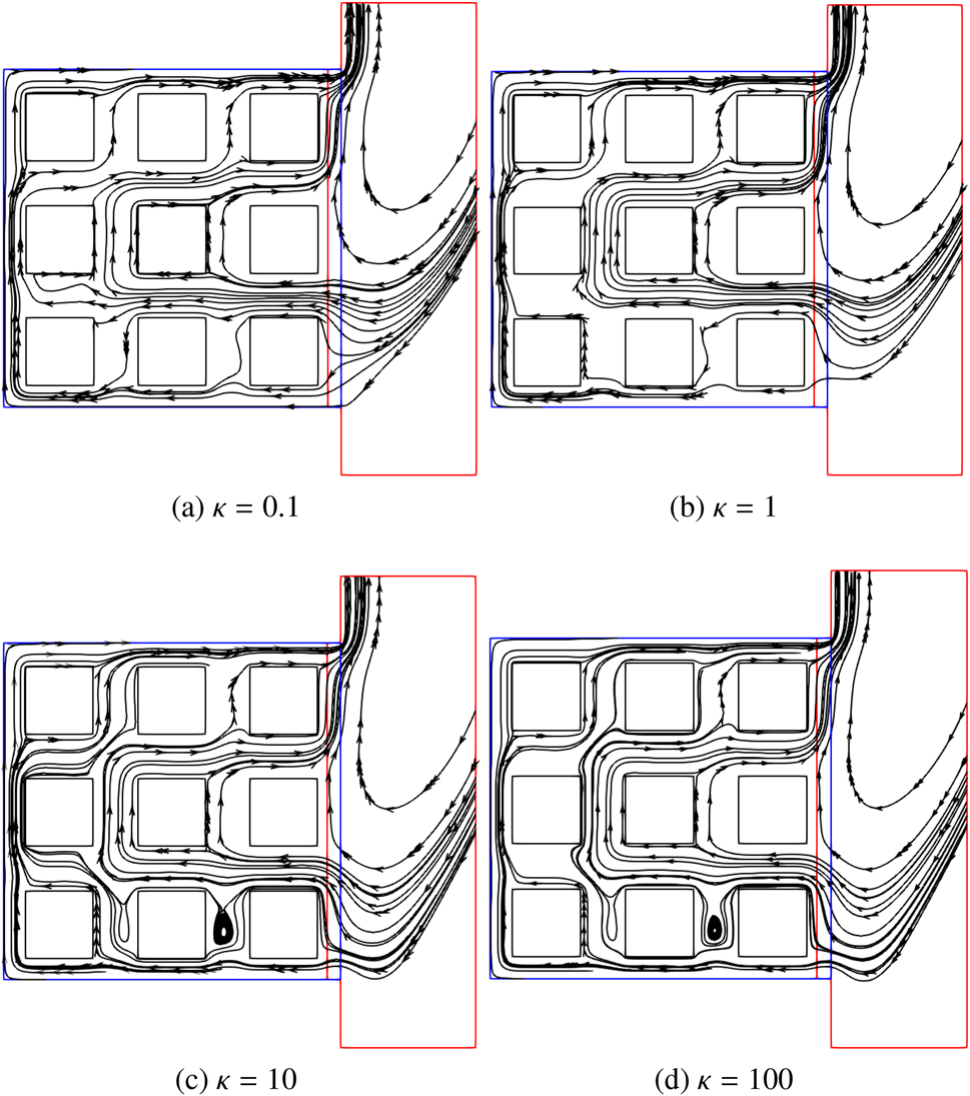
Two-dimensional natural convection in a side open cavity with porous media: local streamline distributions

Additionally, Fig. 19 shows the local velocity field depicted by streamlines at different $\kappa$. The fluid enters the cavity through the bottom gaps between blocks or blocks and wall. Next to the heating wall, the heated fluid rises due to the buoyancy effect, and then it turns right before reaching the top wall. Eventually, the fluid flows through the top gaps between blocks or blocks and wall, and leave the cavity alongside the external vertical wall. The observations are in excellent agreement with the findings in Ref. [50]. In addition, it shows that the streamlines smoothly cross through the coupling interfaces.

Table 7 quantitatively compares the averaged Nusselt numbers on the heated wall obtained by the present coupling model and Ref. [50] for different values of $\kappa$. A maximum relative error of $2.95~\%$ at $\kappa =10$ is obtained.

**Table 7:** Two-dimensional natural convection in a side open cavity with porous media: comparisons of Nusselt number for different ratios of $\kappa$

$\kappa$	Present	Ref. [50]	Relative error
0.1	1.649	1.668	1.14%
1	1.902	1.873	1.55%
10	2.515	2.443	2.95%
100	2.735	2.667	2.55%

Additionally, the dimensionless mass flow rate, $m_{\textrm{in}}$, entering the cavity through the side opening ($x/L=1$) is used to evaluate the accuracy of the coupling scheme, and it is computed as

43 $$\begin{aligned} m_{\textrm{in}} = \left\{ \begin{aligned}&-\int _0^L \frac{u \,L}{\alpha _f} \, \textrm{d}y, ~~~~~~~\text {when}~u<0, \\&~~0, ~~~~~~~~~~~~~~~~~~~~~~~~~\text {when}~u\ge 0. \end{aligned} \right. \end{aligned}$$

Table 8 lists the mass flow rates obtained from our model and Ref. [50], together with the relative errors. It shows the mass flow rate increases with the increase of $\kappa$, proving the improvement of heat transfer inside the cavity. However, our values are smaller, with a relative discrepancy around $8.0~\%$. The mismatch is induced by the different boundary condition on the top, right and bottom boundaries in thermal reservoir since *code_saturne* does not support uncoupled velocity components on the boundary as adopted in Ref. [50].

**Table 8:** Two-dimensional natural convection in a side open cavity with porous media: comparisons of mass flow for for different ratios of $\kappa$

$\kappa$	Present	Ref. [50]	Relative error
0.1	3.802	4.157	8.54%
1	4.359	4.749	8.21%
10	5.237	5.700	8.12%
100	5.483	5.960	8.00%

### Two-dimensional normal plate velocity

**Fig. 20:**
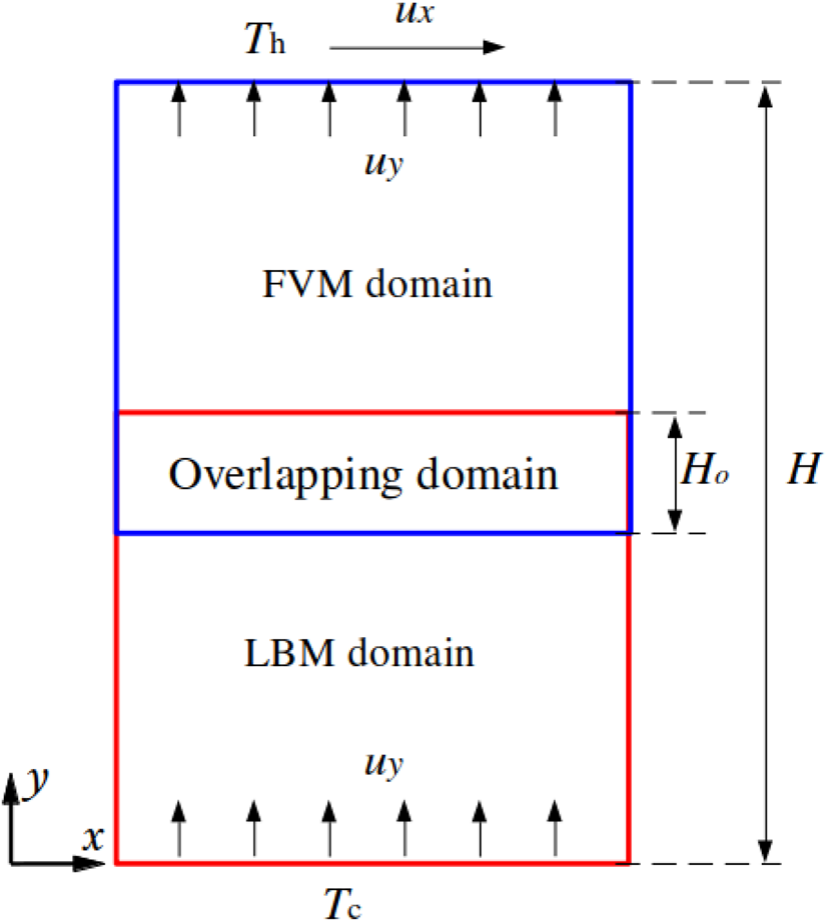
Normal plate velocity: sketch of geometrical setup

The normal plate velocity case is computed to evaluate the numerical performance of the coupling model under the forced convection heat transfer condition. The coupled computational domain is displayed in Fig. 20 where blue and red regions are solved by LBM and FVM, with height 0.55*H* and 0.5*H*, and an overlap of $H_o=0.05H$. Height *H* is discretised with 200 grid points, while only five points are used in the *x* direction. Periodic boundary conditions are applied to the left and right sides. The upper edge moves with a constant uniform horizontal rightward velocity $u_x$ whilst a uniform vertical upward flow with velocity $u_y$ is injected through the bottom wall and withdrawn from the top one. The constant high ($T_h$) and low ($T_c$) temperatures are set at the top and bottom borders with dimensionless units $\theta _h=1.0$ and $\theta _c=0.0$. The fluid is initialised at rest with an averaged temperature $\theta _0 = 0.5$. The fluid kinematic viscosity is set to $\nu =0.01$ and the Reynolds number is defined by $\textrm{Re}={u_y H / \nu } = 10$. Three simulations are carried out by varying the Prandtl number as $\textrm{Pr}= 0.1, 1, 10$. The problem admits analytical solution in the form

44 $$\begin{aligned} \theta = {T-T_c \over T_h-T_c} = {{\textrm{exp}( \mathrm {Re \, Pr} ~y/L) -1} \over {\textrm{exp}(\mathrm {Re \, Pr})-1}}. \end{aligned}$$

**Fig. 21:**
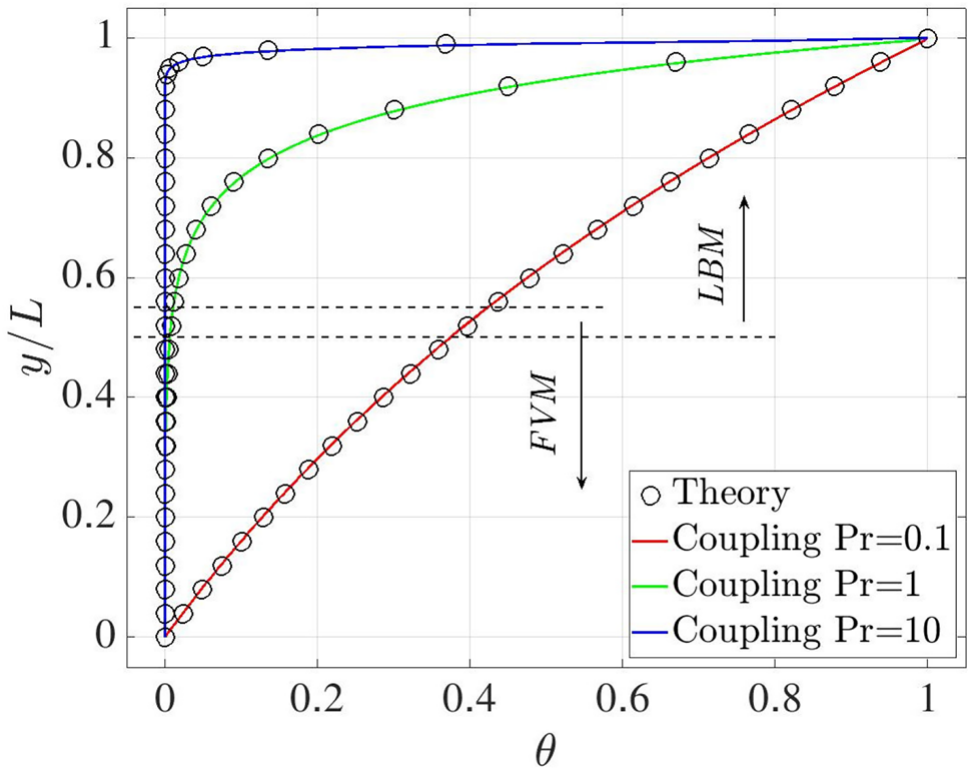
Two-dimensional normal plate velocity: comparisons of temperature distribution between the coupling and analytical solutions for different Pr numbers

With reference to Fig. 21, the present results are in excellent agreement with the analytical solution.

The relative errors between the numerical and analytical solutions calculated by Eq. (38) are reported in Table 9.

**Table 9:** Two-dimensional normal plate velocity: percentage relative discrepancy between present results and analytical predictions

Pr	0.1	1	10
$\epsilon \, [\%]$	0.41	2.00	3.65

### Two-dimensional thermal lid-driven cavity flow

**Fig. 22:**
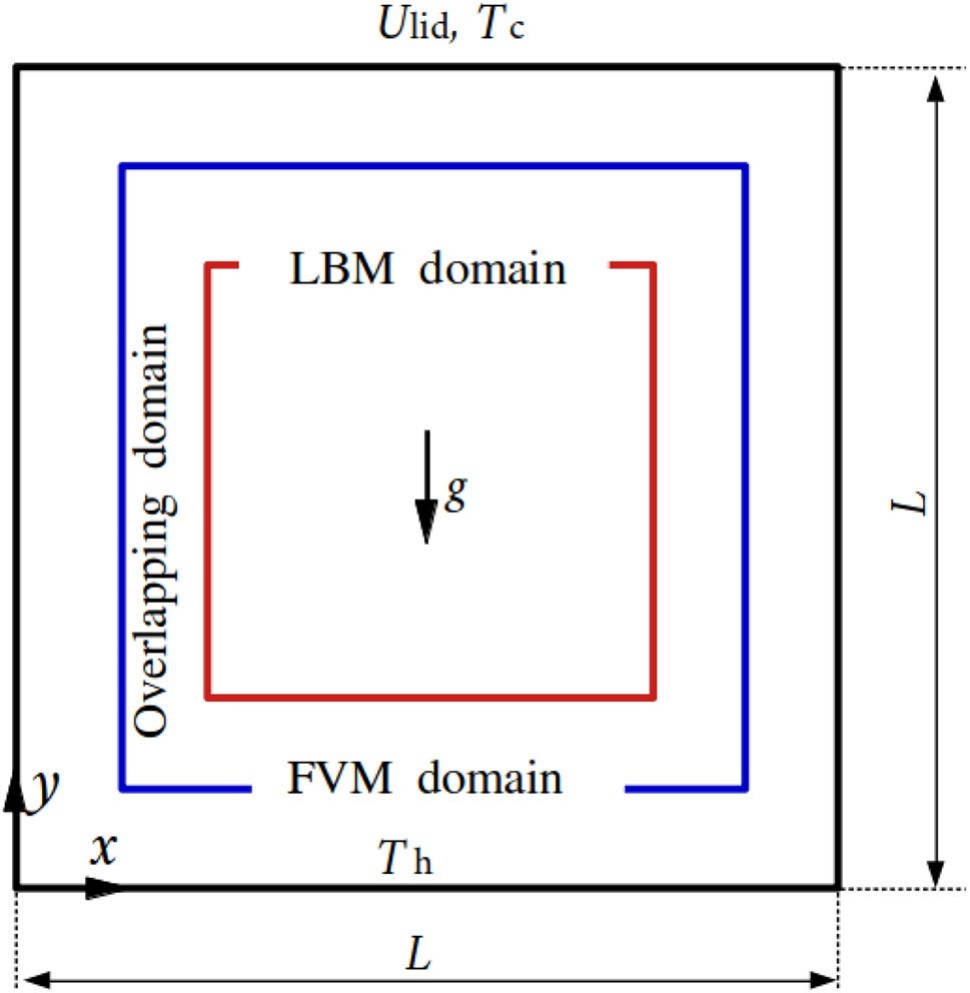
Two-dimensional thermal lid-driven cavity flow: sketch of the computational domain

The thermal lid-driven cavity flow is another popular benchmark case to assess the performance of a numerical tool. Figure 22 describes the computational domain of size $L^2$. The blue square domain of size $(0.66L)^2$ is solved by the LBM while the domain except the red part is simulated by the FVM. A uniform resolution (1/500) is enforced to the entire computational domain, with 10 grid points in the overlapping region, perpendicular to the coupling interfaces. The lid moves to the right with a uniform velocity ($U_{lid}$) while the remaining boundaries are set to no-slip.

In addition, the low ($T_c$) and high ($T_h$) temperatures are enforced on the top and bottom borders with dimensionless units $\theta _c=0.0$ and $\theta _h=1.0$, whilst left and right boundaries adopt the adiabatic condition. The time steps are set equal to 0.02 and 0.002 on the FVM and LBM sides. The problem is governed by the Prantl number with Pr $=\nu /\alpha =0.71$, the Grashof number with Gr $\displaystyle = {g\beta (T_h-T_c) L^3 / \nu ^2} = 10^6$, the Reynolds number with Re $\displaystyle = {U_{lid} L / \nu }$ and the Richardson number Ri $\displaystyle = { \mathrm {Gr / Re^2}}$. Three scenarios are investigated by varying the Richardson number as Ri $= 0.1,~1,~10$. The kinematic viscosity is set to $\nu =4.0\times 10 ^{-5}$ and the thermal diffusivity is $\displaystyle \alpha ={\lambda / \rho c_p}=5.6\times 10 ^{-5}$.

**Fig. 23:**
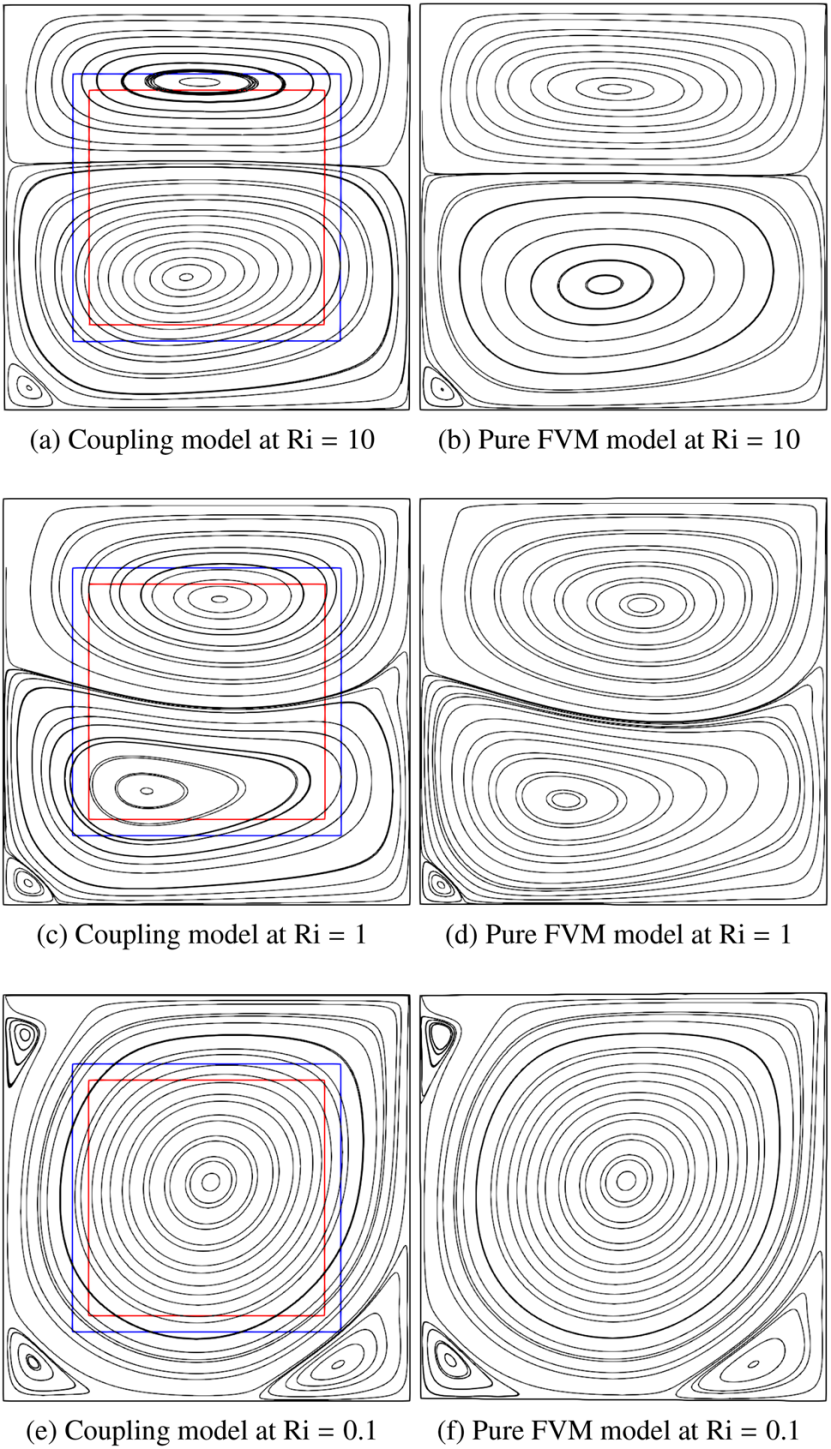
Two-dimensional thermal lid-driven cavity flow: comparisons of streamline distribution between the coupled and pure FVM models

Figure 23 compares the streamline distributions between the coupling framework and pure FVM model using *code_saturne* at Ri = 10, 1 and 0.1, and the results from the pure FVM simulation are selected as the baseline. At Ri $= 10$, two counter-rotating vortices are generated on the upper and lower parts thanks to the interaction of the buoyancy effect because of the unstable temperature gradient and the shear force due to the moving lid. Moreover, the lower vortex exhibits a larger size compared to the upper vortex, as the influence of buoyancy dominates over shear stress at Ri $=10$. The hot and cold fluids converge in the central region, where the densely packed streamlines are observed. A secondary vortex forms in the lower-left corner due to the dominant buoyancy effect. As the Ri number decreases to 1, the shear effect intensifies and balances the buoyancy effect, producing two counter-rotating vortices of comparable strength. However, the buoyancy-induced vortex structure becomes distorted due to the opposing motion of the lid. Notably, the secondary vortex persists in the same location as observed in the Ri $=10$ case. When Ri further decreases to 0.1, the lid-driven effect dominates the entire cavity, forming a primary vortex at the centre and three small secondary vortices near the corners. In addition, the streamlines exhibit smooth continuity across the coupling interfaces, and the vortex structures in the coupled model agree well with those obtained from the pure FVM simulation. This consistency validates the accuracy of velocity coupling scheme.

**Fig. 24:**
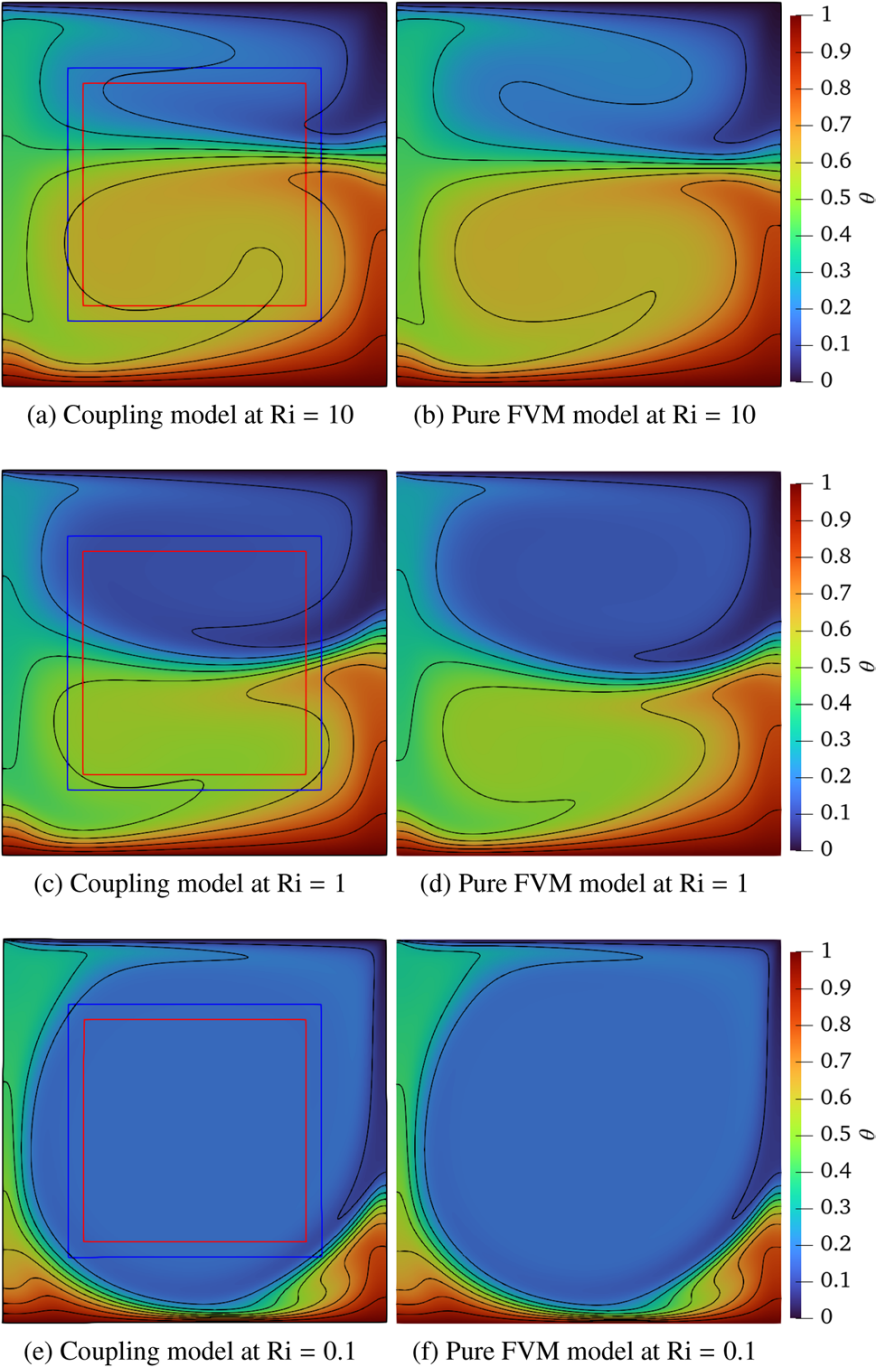
Two-dimensional thermal lid-driven cavity flow: comparisons of temperature field with isotherms between the coupled and pure FVM models

Figure 24 presents the dimensionless temperature distributions with isotherms between the coupled and pure FVM models at Ri $=10$, 1 and 0.1. The isotherms are plotted at constant intervals of $\Delta \theta = 0.1$, denoted by adjacent solid lines. Excellent agreement is observed between the coupled model and pure FVM results, with isotherms demonstrating smooth continuity across coupling interfaces, demonstrating the correctness of temperature coupling scheme. At Ri $=10$ and 1, enhanced thermal mixing in the central region produces steeper temperature gradients due to the interaction between hot and cold fluids. When Ri decreases to 0.1, the lid-driven flow dominates the entire cavity, resulting in cold fluid occupying most of domain. The isotherm distributions reveal particularly steep temperature gradients along the bottom heated wall, attributable to combined shear effects and fluid impingement phenomena.

Table 10 shows the values of Nusselt number computed along the heating wall, comparing results from the pure FVM, coupled model, and Refs [51, 52]. The pure FVM simulations demonstrate closer agreement with reference data. Using the values from Ref. [51] as baseline, our coupled model shows relative errors of 5.60 %, 6.1 % and 3.69 % at Ri $=10$, 1 and 0.1, respectively. This discrepancy primarily stems from the local difference between constant FVM solver density and computed variable density in the LBM boundaries.

**Table 10:** Two-dimensional thermal lid-driven cavity flow: comparisons of Nusselt number on the heating wall at Ri $=10$, 1 and 0.1

Ri	Ref. [51]	Ref. [52]	Pure FVM	Present
10	4.860	4.848	4.862	4.588
1	5.750	5.739	5.758	5.402
0.1	12.161	12.138	12.580	12.610

### Three-dimensional natural convection in a cavity

**Fig. 25:**
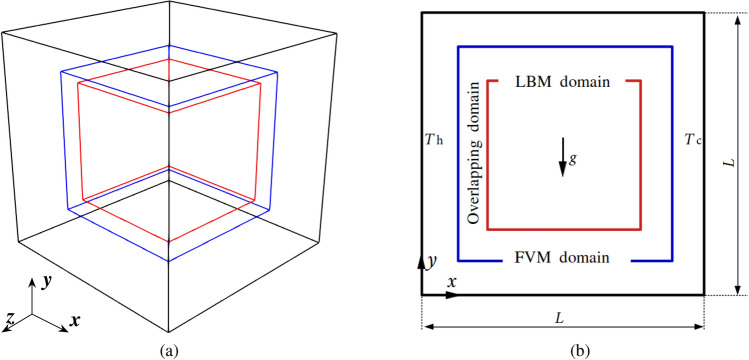
Three-dimensional natural convection: **a** computational domain partition and **b** a cross-section on symmetrical plane

The proposed framework is further validated against the three-dimensional natural convection flow in a cubic cavity. Figure 25 shows the computational domain of size $L^3$ and a cross-section along the symmetry plane located at $z=L/2$. The blue region of size $0.66L^3$ is assigned to the LBM solver, while the rest of the domain except the red part is solved by the FVM. The no-slip condition is enforced for all sides. Constant temperatures corresponding to $\theta _h=1.0$ and $\theta _c=0.0$ are enforced on the planes $x=0$ and $x=L$, respectively, while the other boundaries adopt the adiabatic condition. Two values of the Rayleigh number are investigated, i.e., Ra$=10^5$ and $10^6$.

At Ra $=10^5$, a uniform grid density is applied to the FVM and LBM domains with the resolution of 1/100. In the overlapping region, the distance perpendicular to coupling interface is set to 0.1*L*. The kinematic viscosity and thermal diffusivity are set equal to $1.667\times 10^{-4}$ and $2.347\times 10^{-4}$, respectively, so the Prandtl number is equal to 0.71. The time step on the FVM and LBM sides are 0.05 and 0.01, respectively, so each FVM time step corresponds to 5 sub-iterations on the LBM side.

**Fig. 26:**
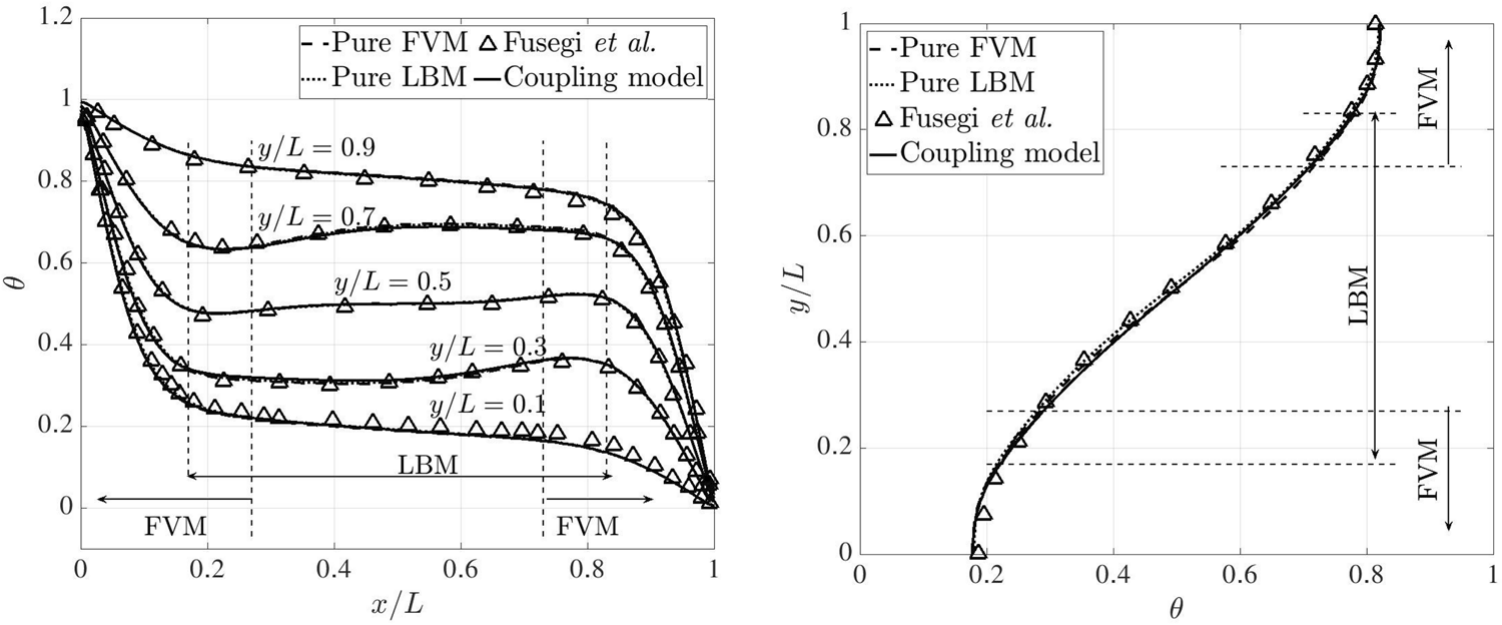
Three-dimensional natural convection: dimensionless temperature distributions along the horizontal (left) and vertical (right) centrelines on the plane $z=0.5L$ at Ra$=10^5$

Figure 26 presents the dimensionless temperature distributions along the horizontal and vertical centrelines on the plane $z=0.5L$, comparing the results obtained from the pure FVM, LBM and reference data [53], where a third-order accurate finite difference approach was adopted. The pure FVM and pure LBM cases are solved using *code_saturne* and LUMA, respectively. Horizontally, five temperature distributions are displayed at $y/L=0.1,~ 0.3,~ 0.5,~ 0.7$ and 0.9. Note that two monitoring lines ($y/L=0.1$ and 0.9) lie completely within the FVM domain. The temperature profiles are very close to the results from pure FVM, LBM and Ref. [53], and the smooth transitions of temperature distribution through the coupling interfaces manifest the correctness of thermal coupling scheme.

**Fig. 27:**
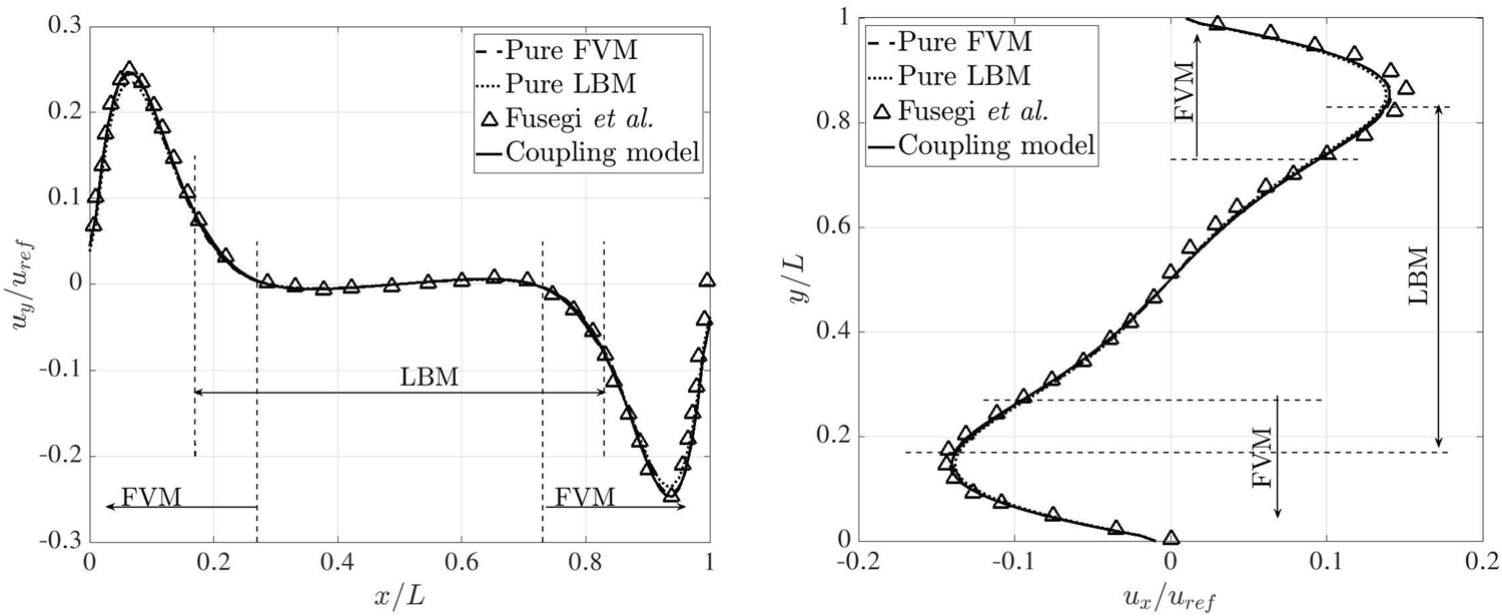
Three-dimensional natural convection: dimensionless velocity distributions along the horizontal (left) and vertical (right) centrelines on the plane of $z=0.5L$ at Ra$=10^5$

Following the above horizontal and vertical lines on the plane $z=0.5L$, Fig. 27 compares the velocity distributions, which are normalised by the reference velocity $u_{\textrm{ref}}=\sqrt{g\beta h \Delta T}$, between the pure FVM, pure LBM, and Ref. [53]. Our present results agree well with those of the pure FVM, LBM and reference. In the overlapping region, the same velocity distributions can be observed in the coupled FVM and LBM regions.

**Fig. 28:**
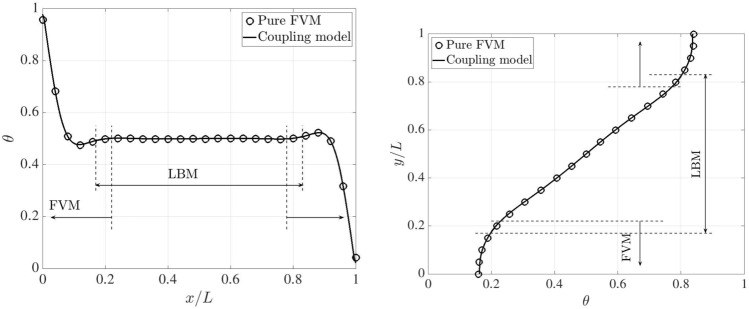
Three-dimensional natural convection: dimensionless temperature distributions along the horizontal (left) and vertical (right) centrelines on the plane of $z=0.5L$ at Ra$=10^6$

For a higher Rayleigh number of $10^6$, the grid resolution is refined to 1/200 on both FVM and LBM sides. In the overlapping region, the distance perpendicular to the coupling interfaces decreases to 0.05*L*. The time steps on the FVM and LBM sides are set to 0.01 and 0.001, respectively. The kinematic viscosity and thermal diffusivity are set to $4.17\times 10^{-4}$ and $5.86\times 10^{-4}$. In this case, the results from the pure FVM computed by *code_saturne* are selected as baseline and used for comparison with those from the coupling model. The variation of temperature across the horizontal and vertical centrelines on the plane $z=0.5L$ are shown in Fig. 28. Notably, the temperature distributions are identical between the pure FVM and coupling model.

**Fig. 29:**
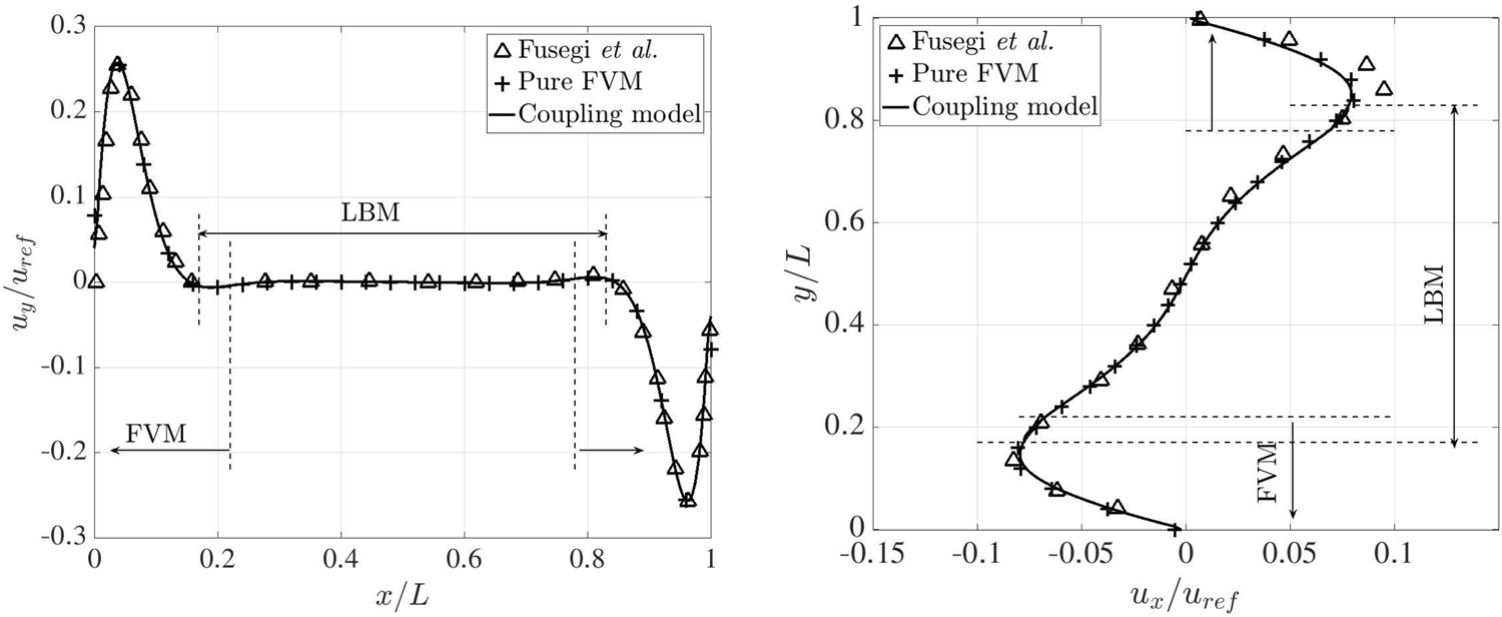
Three-dimensional natural convection: dimensionless velocity distributions along the horizontal (left) and vertical (right) centrelines on the plane of $z=0.5L$ at Ra$=10^6$

Furthermore, Fig. 29 presents a quantitative comparison of the dimensionless velocity distributions along the horizontal and vertical centrelines for the pure FVM, the coupled model, and data in Ref. [53]. Interestingly, our results closely match those of the pure FVM and the reference. For the $u_x/u_{\textrm{ref}}$ profile, a slight deviation is observed near the top boundary between the numerical models and the reference. Nevertheless, the results from both the pure FVM and the coupled model remain physically consistent, as the system’s symmetry naturally leads to symmetrical velocity distributions.

**Fig. 30:**
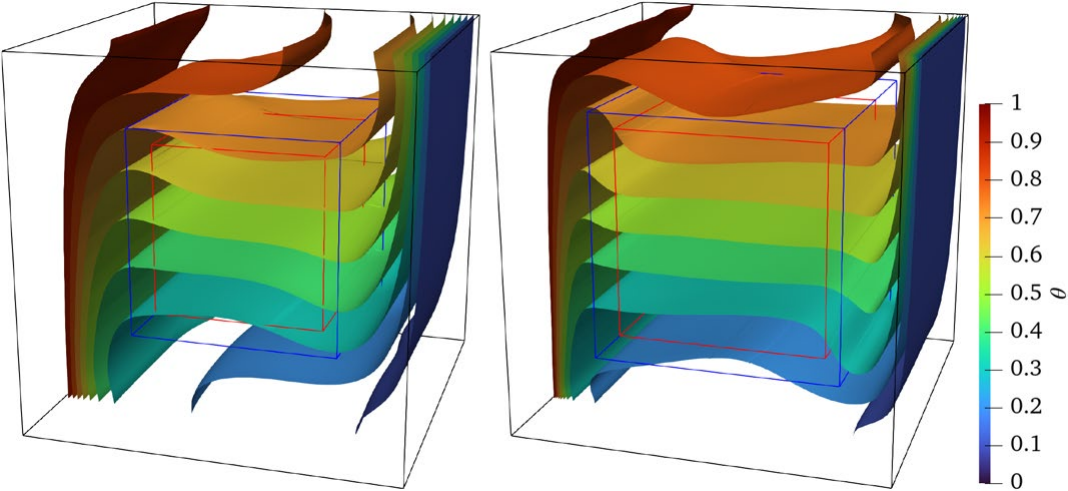
Three-dimensional natural convection: isothermal surfaces at Ra = $10^5$ (left) and $10^6$ (right)

Across the entire coupling domain, Fig. 30 illustrates the isothermal surface distributions at Ra = $10^5$ and $10^6$ with a maintained dimensionless temperature difference of $\Delta \theta = 0.1$ between adjacent surfaces. In both cases, the isothermal surfaces exhibit smooth continuity through the overlapping region, though minor divergences near the coupling interfaces due to the weak compressibility effects inherent in the LBM.

### Rayleigh-Bénard convection with a melting boundary

**Fig. 31:**
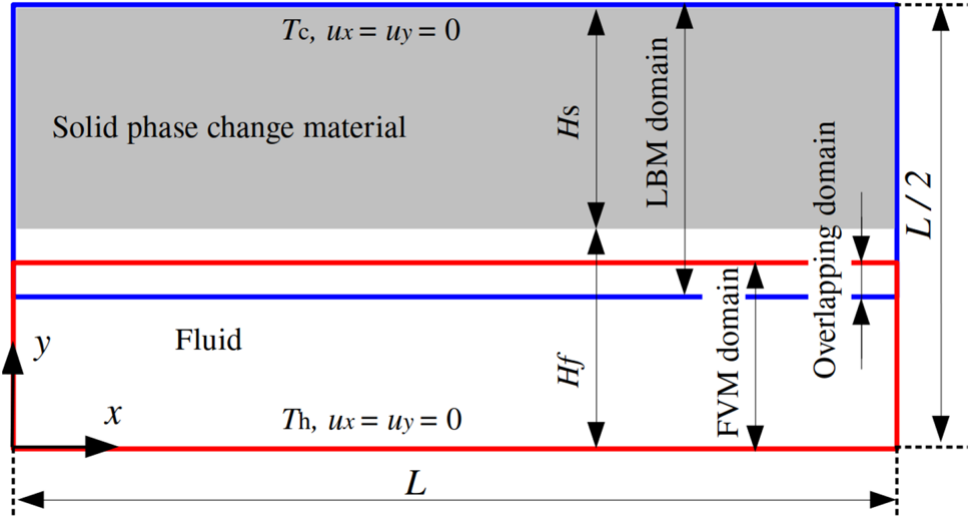
Rayleigh Bénard convection with a melting boundary: sketch of the problem setup

The advantages of the proposed coupled approach are demonstrated in complex melting processes, where the complementary strengths of LBM and FVM are effectively combined. The LBM excels at resolving local phase-change dynamics and associated thermal flows, while the FVM is more suitable for large-scale single-phase thermal flow simulations. To exemplify this concept, Fig. 31 illustrates the two-dimensional computational domain of size $L\times L/2$, with the LBM (blue) and FVM (red) domains occupying $L\times 0.33L$ and $L\times 0.21L$, respectively. The overlapping domain is set to $L\times 0.04L$. Additionally, a consistent grid resolution of *L*/500 is employed in both computational domains to resolve the phase-change dynamics.

The computational domain is initialised with the fluid and solid phases occupying the bottom and top regions, respectively, each with a height of $H_f=H_s=0.25L$. The flow field begins from a quiescent state, with a uniform initial mass density of 1.0 throughout the domain. The temperature field is initially set to a constant value of $\theta _0 = 0.0$. Constant high and low temperatures ($T_h$ and $T_c$) are prescribed over the bottom and top boundaries, respectively, with dimensionless units of $\theta _h=1.0$ and $\theta _c=0.0$, where $\theta _c$ serves as the threshold temperature for solid melting (triggered when $\theta >\theta _c$). Periodic boundary conditions are applied to the left and right boundaries, while no-slip velocity condition is enforced at the top and bottom boundaries. In order to properly account for the phase-change dynamics, additional source terms are incorporated into the momentum and energy Eqs. (1–3) and they read as follows [54, 55]

45 $$\begin{aligned} \partial _t \boldsymbol{u} + \left( \boldsymbol{u} \cdot \boldsymbol{\nabla } \right) \boldsymbol{u}= & -\dfrac{1}{\rho _0} \boldsymbol{\nabla } p + \nu \boldsymbol{\nabla }^2 \boldsymbol{u} + \varepsilon \boldsymbol{g} \beta (T-T_0) - {\chi \over \rho _0} \boldsymbol{u}, \nonumber \\ {\partial _t T} + \boldsymbol{u} \cdot \boldsymbol{\nabla } T= & \boldsymbol{\nabla } \cdot (\alpha \boldsymbol{\nabla } T) - \displaystyle {\Lambda \over c_p }\partial _t \varepsilon , \end{aligned}$$

where $\chi =1-\varepsilon ^2$ is a penalisation factor. $\varepsilon$ represents the liquid fraction ($\varepsilon =1$ for liquid, $\varepsilon =0$ for solid), and the value is initially set to 1 and 0 in the regions of $y \in [0:0.25L]$ and [0.25*L* : 0.5*L*], respectively.. The thermal properties include latent heat $\Lambda$ and the heat capacity $c_p=1$. In addition to the Rayleigh and Prandtl number, the dynamics of this system is also governed by the Stefan number, St = $c_p \Delta T / \Lambda$, where the temperature difference is $\Delta T = T_h - T_c$. In this study the dimensionless parameters are specified as St $=1$, Pr $=1$ and Ra spans four orders of magnitude (i.e., Ra $=10^5$, $10^6$, $10^7$ and $10^8$). The characteristic time scale is defined as $t_0=\sqrt{L/(2 g \beta \Delta T)}$.

**Fig. 32:**
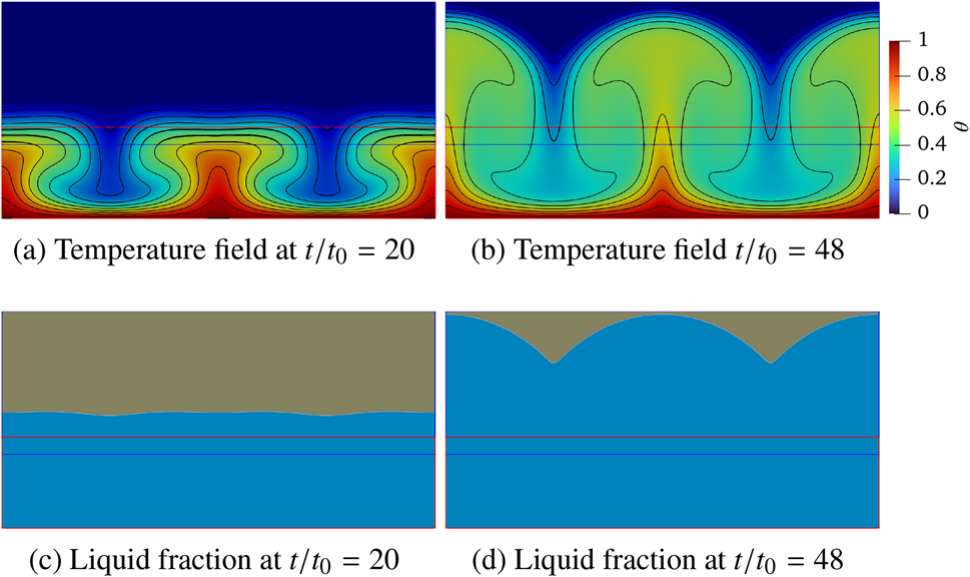
Rayleigh Bénard convection with a melting boundary: temperature and liquid fraction at dimensionless time $t/t_0=20$ and 48 for Ra $=10^5$. Light blue and grey colours correspond to $\varepsilon =1$ (liquid) and $\varepsilon =0$ (solid)

Figure 32 presents the temperature and liquid fraction at times $t/t_0=20$ and 48 by considering Ra $=10^5$. The temperature field reveals the formation of two Bénard convection cells, driven by buoyancy forces under gravity, which induce a wavy melting front characteristic of symmetric convection regimes. These results align with the results shown by De Rosis & Giustini [54], where the LBM was adopted to perform the numerical simulations.

**Fig. 33:**
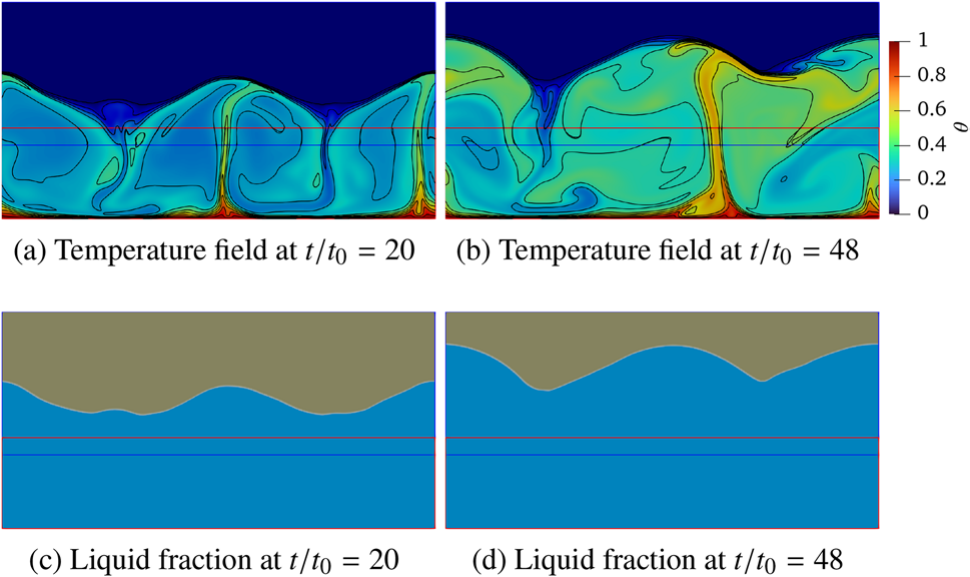
Rayleigh Bénard convection with a melting boundary: temperature and liquid fraction at dimensionless time $t/t_0=20$ and 48 for Ra $=10^8$. Light blue and grey colours correspond to $\varepsilon =1$ (liquid) and $\varepsilon =0$ (solid)

As shown in Fig. 33, the temperature field exhibits unsteady behaviour at the higher Ra number equal to $10^8$, resulting in a significantly more irregular melting front morphology. Despite minor local deviations, the isotherms maintain consistent distribution across the overlapping region, demonstrating once again the robustness of the present methodology under unsteady flow conditions.

To quantitatively evaluate the melting dynamics, Fig. 35 presents the temporal evolution of the domain-averaged liquid fraction, *r*, which is defined as

46 $$\begin{aligned} r(t) = \frac{1}{L \times L/2}\sum _{\boldsymbol{x}} \varepsilon (\boldsymbol{x},t). \end{aligned}$$

**Fig. 34:**
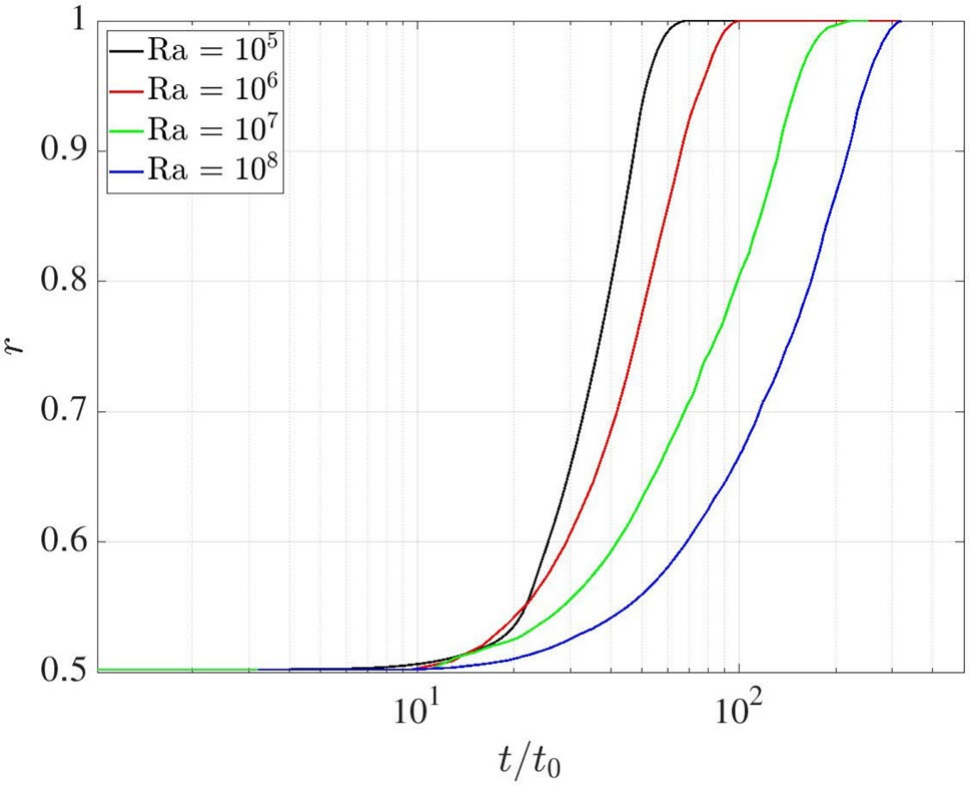
Rayleigh Bénard convection with a melting boundary: time evolution of the liquid fraction at Ra $=10^5$, $10^6$, $10^7$ and $10^8$

**Fig. 35:**
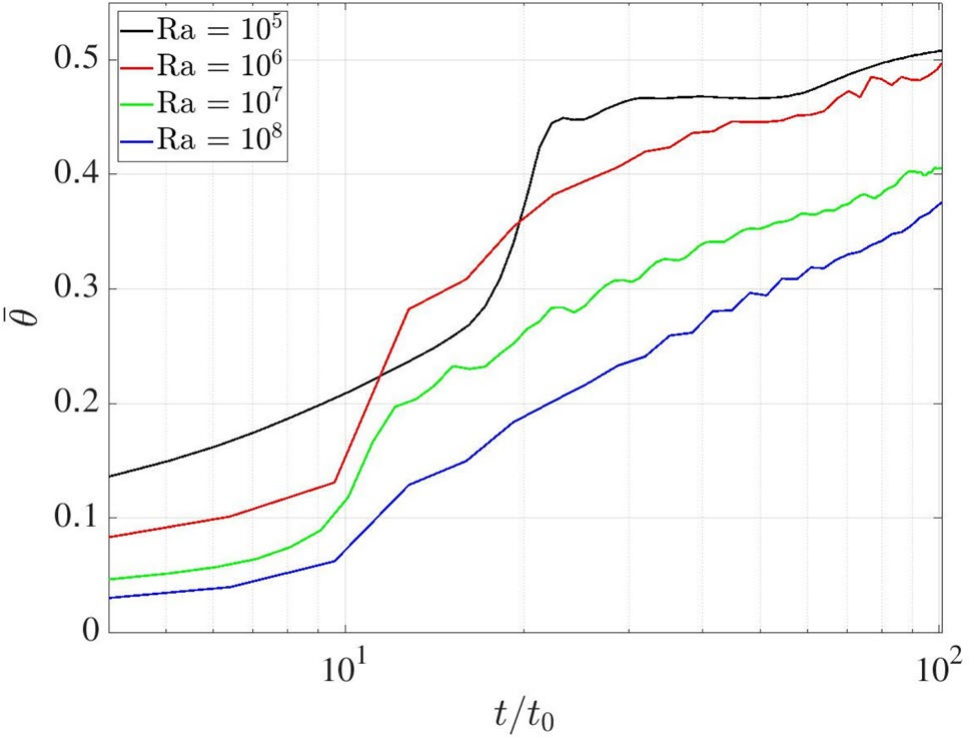
Rayleigh Bénard convection with a melting boundary: time evolution of the averaged temperature in fluid phase at Ra $=10^5$, $10^6$, $10^7$ and $10^8$

Our findings reveal a distinct transition from an initially smooth stage ($t/t_0<10$) to a sharp change in the melting behaviour. Notably, the rate of phase change, as characterised by the slope of the liquid fraction curve, exhibits an inverse relationship with the Ra number, that is, the higher Ra values correspond to reduced melting rates. This phenomenon can be attributed to the enhanced convective heat transfer at higher Ra (Fig. 34). Since the temperature acts as the primary factor governing the melting process, the variation of average temperature ($\bar{\theta }$) in the fluid phase, representing the degree of mixing between the hot and cold fluids, is shown in Fig. 35 for different Ra numbers. Similar to the phase-change behaviour, consistent trends are observed in the average temperature evolution. Higher Ra conditions enhance fluid mixing and lead to a reduction in the the average temperature, thereby suppressing the overall phase-change process.

## Conclusions and future work

We have developed a robust coupling framework between the finite volume method and the lattice Boltzmann method for thermal flow simulations, employing a central-moments-based collision operator and advanced reconstruction techniques. The framework is implemented through the *code_saturne* and LUMA solvers, integrated using the Parallel Location Exchange (PLE) coupling library for efficient communication between FVM and LBM solvers.

Quantitative validation against seven benchmark problems, including both diffusive and convective regimes, complex geometries, and phase change, demonstrates the accuracy and versatility of the proposed approach. Notably, the use of central moments in both velocity and temperature fields reduces interface discontinuities and improves convergence: for example, in natural convection at $\text {Ra} = 10^6$, temperature residuals dropped by two orders of magnitude compared to BGK-based implementations. The reconstructed temperature and velocity profiles show excellent agreement with analytical and reference data across all cases.

Overall, the method constitutes a significant advance in the coupling of LBM and FVM, enabling accurate and efficient multiscale thermal flow modelling. Future work will focus on exploring high-order temporal interpolation scheme and extending the domain-decomposition-based framework to moving boundaries and phase-change-driven flows characteristic of realistic industrial applications.

## Data Availability

https://github.com/yangzhou-10/code_saturne-LUMA-coupling

## Appendix: Raw moment formulation

According to the Refs. [41, 42], the transformation matrix $\textbf{T}$ can be written as

47 $$\begin{aligned} \textbf{T} = \textbf{N} \textbf{M}, \end{aligned}$$

where the transformation matrix $\textbf{M}$ read as

48 $$\begin{aligned} {\textbf{M}} = \left[ \begin{array}{c} \langle |\boldsymbol{c}_i|^0| \\ \langle {c}_{ix}|\\ \langle {c}_{iy}| \\ \langle {c}_{iz}| \\ \langle {c}_{ix}^2+ {c}_{iy}^2 + {c}_{iz}^2 | \\ \langle {c}_{ix}^2- {c}_{iy}^2 | \\ \langle {c}_{iy}^2- {c}_{iz}^2|\\ \langle {c}_{ix} {c}_{iy}| \\ \langle {c}_{ix} {c}_{iz} | \\ \langle {c}_{iy} {c}_{iz}| \\ \langle {c}_{ix}^2{c}_{iy}|\\ \langle {c}_{ix}{c}_{iy}^2| \\ \langle {c}_{ix}^2{c}_{iz}|\\ \langle {c}_{ix}{c}_{iz}^2| \\ \langle {c}_{iy}^2{c}_{iz}| \\ \langle {c}_{iy}{c}_{iz}^2| \\ \langle {c}_{ix}^2{c}_{iy}^2| \\ \langle {c}_{ix}^2{c}_{iz}^2| \\ \langle {c}_{iy}^2{c}_{iy}^2| \end{array} \right] . \end{aligned}$$

Therefore, the present central-moments-based framework can be considered as a general multiple-relaxation-time LBM, where the classical raw-moments-based MRT is recovered if $\textbf{T}=\textbf{M}$, leading to $\textbf{N}=\textbf{I}$. We further note that the transformation matrix $\textbf{M}$ transforms the distribution functions into the raw moments, while the shift matrix $\textbf{N}$ transforms the raw moments into the CMs. While not strictly necessary, this two-step approach allows for an easier implementation of the central-moments-based LBM. The post-collision thermal distribution functions can be rewritten as

49 $$\begin{aligned} |g_i^{\star }\rangle = \textbf{T}^{-1} |k_{i, T}^{\star } \rangle = \textbf{M}^{-1} \textbf{N}^{-1} |k_{i, T}^{\star } \rangle , \end{aligned}$$

where the post-collision raw moments $|r_{i,T}^*\rangle = [r_{0,T}^*,..., r_{i,T}^*,...,r_{18,T}^*]^{\top }$ are evaluated as

50 $$\begin{aligned} |r_{i, T}^{\star }\rangle = \textbf{N}^{-1} |k_{i, T}^{\star } \rangle . \end{aligned}$$

which are equal to

51 $$\begin{aligned} r_{0,T}^{\star }&= T, \nonumber \\ r_{1,T}^{\star }&= k_{1,T}^{\star } + T u_x, \nonumber \\ r_{2,T}^{\star }&= k_{2,T}^{\star } + T u_y, \nonumber \\ r_{3,T}^{\star }&= k_{3,T}^{\star } + T u_z, \nonumber \\ r_{4,T}^{\star }&= k_{4,T}^{\star } + 2 u_x k_{1,T}^{\star } + 2 u_y k_{2,T}^{\star } + 2 u_z k_{3,T}^{\star } + T (u_x^2 + u_y^2 + u_z^2), \nonumber \\ r_{5,T}^{\star }&= 2 u_x k_{1,T}^{\star } - 2 u_y k_{2,T}^{\star } + T (u_x^2 - u_y^2), \nonumber \\ r_{6,T}^{\star }&= 2 u_y k_{2,T}^{\star } - 2 u_z k_{3,T}^{\star } + T (u_y^2 - u_z^2), \nonumber \\ r_{7,T}^{\star }&= u_x k_{2,T}^{\star } + u_y k_{1,T}^{\star } + T u_x u_y, \nonumber \\ r_{8,T}^{\star }&= u_x k_{3,T}^{\star } + u_z k_{1,T}^{\star } + T u_x u_z, \nonumber \\ r_{9,T}^{\star }&= u_y k_{3,T}^{\star } + u_z k_{2,T}^{\star } + T u_y u_z, \nonumber \\ r_{10,T}^{\star }&= u_x^2 k_{2,T}^{\star } + 2 u_x u_y k_{1,T}^{\star } + u_y c_s^2 k_{4,T}^{\star } + T u_x^2 u_y, \nonumber \\ r_{11,T}^{\star }&= u_y^2 k_{1,T}^{\star } + 2 u_x u_y k_{2,T}^{\star } + u_x c_s^2 k_{4,T}^{\star } + T u_x u_y^2, \nonumber \\ r_{12,T}^{\star }&= u_x^2 k_{3,T}^{\star } + 2 u_x u_z k_{1,T}^{\star } + u_z c_s^2 k_{4,T}^{\star } + T u_x^2 u_z, \nonumber \\ r_{13,T}^{\star }&= u_z^2 k_{1,T}^{\star } + 2 u_x u_z k_{3,T}^{\star } + u_x c_s^2 k_{4,T}^{\star } + T u_x u_z^2, \nonumber \\ r_{14,T}^{\star }&= u_y^2 k_{3,T}^{\star } + 2 u_y u_z k_{2,T}^{\star } + u_z c_s^2 k_{4,T}^{\star } + T u_y^2 u_z, \nonumber \\ r_{15,T}^{\star }&= u_z^2 k_{2,T}^{\star } + 2 u_y u_z k_{3,T}^{\star } + u_y c_s^2 k_{4,T}^{\star } + T u_y u_z^2, \nonumber \\ r_{16,T}^{\star }&= k_{16,T}^{\star } + k_{4,T}^{\star } (c_s^2 u_x^2 + c_s^2 u_y^2) + T u_x^2 u_y^2 + 2 u_x u_y^2 k_{1,T}^{\star } + \nonumber \\&\quad 2 u_x^2 u_y k_{2,T}^{\star }, \nonumber \\ r_{17,T}^{\star }&= k_{17,T}^{\star } + k_{4,T}^{\star } (c_s^2 u_x^2 + c_s^2 u_z^2) + T u_x^2 u_z^2 + 2 u_x u_z^2 k_{1,T}^{\star } + \nonumber \\&\quad 2 u_x^2 u_z k_{3,T}^{\star }, \nonumber \\ r_{18,T}^{\star }&= k_{18,T}^{\star } + k_{4,T}^{\star } (c_s^2 u_y^2 + c_s^2 u_z^2) + T u_y^2 u_z^2 + 2 u_y u_z^2 k_{2,T}^{\star } + \nonumber \\&\quad 2 u_y^2 u_z k_{3,T}^{\star }. \end{aligned}$$

Finally, the post-collision populations are constructed as

52 $$\begin{aligned} |g_i^{\star }\rangle = \textbf{M}^{-1} |r_{i, T}^{\star } \rangle , \end{aligned}$$

*i.e.,*

53 $$\begin{aligned} g_0^{\star }&= r_{0,T}^{\star }-r_{4,T}^{\star }+r_{16,T}^{\star }+r_{17,T}^{\star }+r_{18,T}^{\star }, \nonumber \\ g_1^{\star }&= \left( r_{4,T}^{\star }+2 r_{5,T}^{\star }+r_{6,T}^{\star }\right) c_s^2/2-\nonumber \\&\,\left( -r_{1,T}^{\star }+r_{11,T}^{\star }+r_{13,T}^{\star }+r_{16,T}^{\star }+r_{17,T}^{\star }\right) /2, \nonumber \\ g_2^{\star }&= \left( r_{4,T}^{\star }+2 r_{5,T}^{\star }+r_{6,T}^{\star }\right) c_s^2/2+\nonumber \\&\,\left( r_{11,T}^{\star }+r_{13,T}^{\star }-r_{1,T}^{\star }-r_{16,T}^{\star }-r_{17,T}^{\star }\right) /2, \nonumber \\ g_3^{\star }&= \left( r_{4,T}^{\star }-r_{5,T}^{\star }+r_{6,T}^{\star }\right) c_s^2/2-\nonumber \\&\,\left( -r_{2,T}^{\star }+r_{10,T}^{\star }+r_{15,T}^{\star }+r_{16,T}^{\star }+r_{18,T}^{\star }\right) /2, \nonumber \\ g_4^{\star }&= \left( r_{4,T}^{\star }-r_{5,T}^{\star }+r_{6,T}^{\star }\right) c_s^2/2-\nonumber \\&\,\left( r_{2,T}^{\star }-r_{10,T}^{\star }-r_{15,T}^{\star }+r_{16,T}^{\star }+r_{18,T}^{\star }\right) /2, \nonumber \\ g_5^{\star }&= \left( r_{4,T}^{\star }-r_{5,T}^{\star }-2 r_{6,T}^{\star }\right) c_s^2/2-\nonumber \\&\,\left( -r_{3,T}^{\star }+r_{12,T}^{\star }+r_{14,T}^{\star }+r_{17,T}^{\star }+r_{18,T}^{\star }\right) /2, \nonumber \\ g_6^{\star }&= \left( r_{4,T}^{\star }-r_{5,T}^{\star }-2 r_{6,T}^{\star }\right) c_s^2/2-\nonumber \\&\,\left( r_{3,T}^{\star } -r_{12,T}^{\star }-r_{14,T}^{\star }+r_{17,T}^{\star }+r_{18,T}^{\star }\right) /2, \nonumber \\ g_7^{\star }&= \left( r_{7,T}^{\star }+r_{10,T}^{\star }+r_{11,T}^{\star }+r_{16,T}^{\star }\right) /4, \nonumber \\ g_8^{\star }&= \left( r_{7,T}^{\star }+r_{16,T}^{\star }-r_{10,T}^{\star }-r_{11,T}^{\star }\right) /4, \nonumber \\ g_9^{\star }&= \left( r_{11,T}^{\star }+r_{16,T}^{\star }-r_{7,T}^{\star }-r_{10,T}^{\star }\right) /4, \nonumber \\ g_{10}^{\star }&= \left( r_{10,T}^{\star }+r_{16,T}^{\star }-r_{7,T}^{\star }-r_{11,T}^{\star }\right) /4, \nonumber \\ g_{11}^{\star }&= \left( r_{8,T}^{\star }+r_{12,T}^{\star }+r_{13,T}^{\star }+r_{17,T}^{\star }\right) /4, \nonumber \\ g_{12}^{\star }&= \left( r_{8,T}^{\star }+r_{17,T}^{\star }-r_{12,T}^{\star }-r_{13,T}^{\star }\right) /4, \nonumber \\ g_{13}^{\star }&= \left( r_{13,T}^{\star }+r_{17,T}^{\star }-r_{8,T}^{\star }-r_{12,T}^{\star }\right) /4, \nonumber \\ g_{14}^{\star }&= \left( r_{12,T}^{\star }+r_{17,T}^{\star }-r_{8,T}^{\star }-r_{13,T}^{\star }\right) /4, \nonumber \\ g_{15}^{\star }&= \left( r_{9,T}^{\star }+r_{14,T}^{\star }+r_{15,T}^{\star }+r_{18,T}^{\star }\right) /4, \nonumber \\ g_{16}^{\star }&= \left( r_{9,T}^{\star }+r_{18,T}^{\star }-r_{14,T}^{\star }-r_{15,T}^{\star }\right) /4, \nonumber \\ g_{17}^{\star }&= \left( r_{15,T}^{\star }+r_{18,T}^{\star }-r_{9,T}^{\star }-r_{14,T}^{\star }\right) /4, \nonumber \\ g_{18}^{\star }&= \left( r_{14,T}^{\star }+r_{18,T}^{\star }-r_{9,T}^{\star }-r_{15,T}^{\star }\right) /4. \end{aligned}$$
